# A four-in-one replicase integrating key enzymatic activities for DNA replication

**DOI:** 10.1093/nar/gkaf542

**Published:** 2025-06-23

**Authors:** Yuxin Zhang, Xueling Lu, Bin Zhu, Fengtao Huang

**Affiliations:** Key Laboratory of Molecular Biophysics, the Ministry of Education, College of Life Science and Technology, Huazhong University of Science and Technology, Wuhan, Hubei 430074, China; Key Laboratory of Molecular Biophysics, the Ministry of Education, College of Life Science and Technology, Huazhong University of Science and Technology, Wuhan, Hubei 430074, China; Key Laboratory of Molecular Biophysics, the Ministry of Education, College of Life Science and Technology, Huazhong University of Science and Technology, Wuhan, Hubei 430074, China; Shenzhen Huazhong University of Science and Technology Research Institute, Shenzhen 518063, China; Key Laboratory of Molecular Biophysics, the Ministry of Education, College of Life Science and Technology, Huazhong University of Science and Technology, Wuhan, Hubei 430074, China

## Abstract

DNA replication is a fundamental process in all living organisms. As the most diverse and abundant biological entities on Earth, bacteriophages may utilize unconventional methods for genome replication. In this study, we identified a novel DNA replicase, GP55, from *lactococcal* phage 1706. GP55 comprises a helicase domain, a distinctive archaeo-eukaryotic primase domain, and a family B DNA polymerase domain, collectively exhibiting helicase, primase, and DNA polymerase activities, along with intrinsic 3′–5′ exonuclease activity. Notably, the helicase activity of GP55 is UTP/dTTP-dependent rather than ATP-dependent and facilitates strand displacement during DNA synthesis. GP55 exhibits a unique primase activity, recognizing specific but less stringent DNA sequences and preferring GTP for the initiation of RNA primer synthesis. Additionally, a newly identified α-helix domain, composed of two pairs of parallel α-helices, was found to be essential for its primase activity. The multiple activities enable GP55 to efficiently synthesize DNA *de novo* in the presence of dNTPs and NTPs. This study reveals a concise strategy employed by bacteriophages for genome replication using multifunctional replicases.

## Graphical Abstract

**Graphical Abstract:**
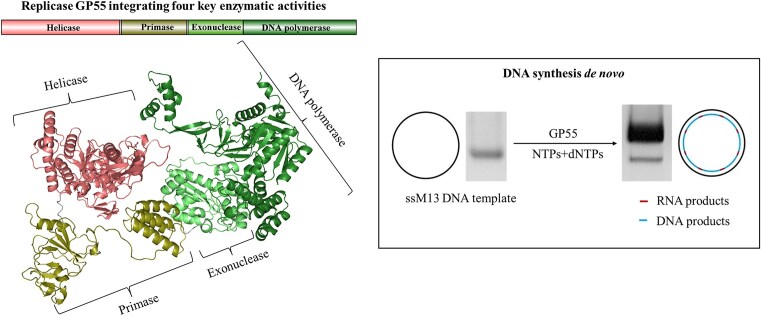

## Introduction

DNA replication is a crucial and multistep process across all domains of life, typically involving various enzymes such as DNA polymerases, primases, and helicases. Among these, DNA polymerases play a key role in DNA replication. However, in cellular organisms, including eukaryotes, archaea, and bacteria, DNA polymerases generally lack the ability to synthesize DNA *de novo* [1]. They rely on primases to generate short RNA primers [1]. Exceptions are found in mobile genetic elements (MGEs) and viruses. For instance, PrimPols, a class of primases in the archaeo-eukaryotic primase (AEP) superfamily, have been identified as replicases capable of *de novo* synthesis for plasmid pRN1 and phage NrS-1 genomes [2–4]; certain family B DNA polymerases (PolBs) from self-replicating MGEs exhibit primer-independent DNA synthesis activities [5]; and a putative family A DNA polymerase, CCPol, encoded by staphylococcal MGE, acquires primase activity by tightly binding to the adjacent small protein MP [6]. However, primer synthesis by primase and primer extension by DNA polymerase are distinct processes [3], posing challenges for simultaneous primase and DNA polymerase activities within a single replicase. Our investigations into the PrimPol (NrSPol) from the NrS-1 phage have revealed conflicts between the primase and DNA polymerase activity within the same catalytic domain [7].

Bacteriophages, the most abundant entities on Earth, exhibit vast genetic diversity. Consequently, the DNA replication systems in bacteriophages are considerably more diverse than those found in cellular organisms [8]. The DNA replication systems of bacteriophages, such as bacteriophages T7, T4, and phi29, are extensively studied as models for DNA replication, due to their simplicity and efficiency [9–12]. The DNA polymerases derived from these bacteriophages serve as essential tools in molecular biology. However, the biochemically characterized DNA replication systems in viruses may represent just the tip of an iceberg. Bioinformatics analysis revealed the presence of diverse, uncharacterized proteins containing DNA polymerase domains in various bacteriophages [8]. Characterizing these novel replicases may unveil unconventional genome replication mechanisms and may introduce new reagents for molecular biology.

In this study, our focus is on the putative ORF55-encoded helicase (GP55) from lactococcal phage 1706 of the *Siphoviridae* family, originally isolated from the failed manufacture of a soft cheese [13]. Phage 1706 has a linear double-stranded DNA (dsDNA) genome of 55 597 bp with 33.7% G + C content and 76 predicted open reading frames (ORFs) [13]. Except for GP55 and the single-stranded binding protein (GP66), its genome does not encode any other DNA replication-related proteins. While GP67 is a putative helicase, it shows homology to RNA helicases. GP55, a large protein containing 1317 amino acid residues, exhibits a unique domain architecture distinct from all presently characterized replication-associated proteins. Our findings demonstrate that GP55 is a multifunctional replicase harboring helicase activity, DNA polymerase activity, 3′–5′ exonuclease activity, and RNA primer synthesis activity in a single protein.

## Materials and methods

### Plasmid construction

The original GP55 gene sequence (protein ID: YP_001828703), the codon-optimized GP55 gene DNA sequence, and the amino acid sequence of the full-length GP55 studied are listed in Supplementary Table S1. The codon-optimized GP55 gene was ordered from GenScript (Nanjing, China) and cloned into expression vector pET28a with an N-terminal 6xHis-tag. GP55 mutants were constructed by whole-plasmid polymerase chain reaction.

### Protein expression and purification

The plasmid for GP55 expression was transformed into *Escherichia coli* BL21(DE3) cells, which were grown overnight at 37°C in LB medium containing 50 μg/ml kanamycin. The cultures were then transferred into auto-induction medium (ZYM-5052) [14] containing 50 μg/ml kanamycin at a ratio of ∼1:100. When the OD_600_ reached ∼2.0, the cultures were transferred to 16°C and grown for another 16 h. The cells were harvested and the cell pellets were resuspended in lysis buffer (20 mM Tris–HCl, pH 8.0, 300 mM NaCl, 20 mM imidazole, 1 mM tris(2-carboxyethyl)phosphine hydrochloride (TCEP)). Cells were lysed by sonication on ice and centrifuged at 13 000 rpm for 40 min at 4°C. The resulting supernatant was collected, and His-tagged GP55 was purified using Ni-NTA resin (Qiagen) and a gravity column. Resin was washed with wash buffer (lysis buffer supplemented to 50 mM imidazole and 500 mM NaCl), and then eluted with elution buffer (20 mM Tris–HCl, pH 8.0, 100 mM NaCl, 200 mM imidazole, and 1 mM TCEP). The eluted protein was concentrated by Millipore Amicon Ultra-15 (30 000 MWCO) and further purified by gel filtration chromatography on a Superdex 200 Increase 10/300 GL column (Cytiva Life Sciences). Fractions containing GP55 were collected and dialyzed twice at 4°C against storage buffer [50 mM Tris–HCl, pH 7.5, 100 mM NaCl, 0.1 mM Ethylenediaminetetraacetic acid (EDTA), 1 mM Dithiothreitol (DTT), 0.1% Triton X-100, and 50% glycerol]. The purified proteins were analyzed by Sodium dodecyl sulphate–polyacrylamide gel electrophoresis (SDS–PAGE) with Coomassie staining, and the protein concentrations were determined by the Bradford method.

### SEC–MALS

The molecular sizes of GP55 and ΔN(1–439) were analyzed by SEC–MALS (size exclusion chromatography coupled to multi-angle light scattering). Both proteins were purified using Ni-NTA resin (Qiagen) as described above. The purified proteins were then loaded onto a Superdex 200 Increase 10/300 GL column (Cytiva Life Sciences) pre-equilibrated with a buffer (20 mM Tris–HCl, pH 8.0, 100 mM NaCl, 1 mM TCEP), at a flow rate of 0.5 ml/min. Light scattering signals were monitored using a Wyatt Dawn Heleos II detector (Wyatt Technology). Data collection and analysis were performed with ASTRA 6 software (Wyatt Technology).

### Primer extension assays

A 16-nt primer (5′-CATGTCAGGGTCTTCA-3′) with 5′-^32^P label was prepared using T4 Polymerase Kinase (New England Biolabs) and [γ-^32^P]ATP (PerkinElmer), and then annealed to a 36-nt template (5′-GAGATCCTATCGAGTAGCTCTGAAGACCCTGACATG-3′). Assays (10 μl final volume) containing 20 mM Tris–Ac (pH 7.9), 50 mM KAc, 10 mM Mg(Ac)_2_, 0.1 mg/ml BSA, 50 nM 5′-labeled primer/template duplex, indicated concentrations of dNTPs, and indicated amounts of GP55 or its mutants were performed at 37°C for indicated time. The reactions were terminated by adding 5 μl of 95% formamide dye containing 20 mM EDTA and heated for 3 min at 90°C. The samples were analyzed by 10% denaturing PAGE containing 7 M urea.

### Strand displacement assays

Two DNA substrates (Substrate A and Substrate B) were used to study the strand displacement activity of GP55. A 65-nt template (5′- CATGTCAGGGTCTTCAGAGCTACTCGATAGGATCTCGGAGAAAAAGTCCCATAGATTCGAAACTT-3′) was annealed to a 5′-FAM-labeled 20-nt primer (5′- AAGTTTCGAATCTATGGGAC-3′) and a 40-nt downstream blocking DNA (5′-CTCCGAGATCCTATCGAGTAGCTCTGAAGACCCTGACATG-3′) or a 60-nt downstream blocking DNA (5′-AAAAAAAAAAAAAAAAAAAACTCCGAGATCCTATCGAGTAGCTCTGAAGACCCTGACATG-3′) to create Substrate A and Substrate B (with a 20-nt overhang). Reactions (10 μl) containing 20 mM Tris–Ac (pH 7.9), 50 mM KAc, 10 mM Mg(Ac)_2_, 0.1 mg/ml BSA, 100 nM Substrate A or Substrate B, 0.5 mM dNTPs, and 50 nM GP55 or ΔN(1–439) were incubated at 37°C for 10 min. The reactions were terminated by adding 10 μl of 2× RNA loading dye (New England Biolabs) and incubating at 95°C for 5 min. T4 and Phi29 DNA polymerases were used as negative and positive controls, respectively. The samples were analyzed by 12% denaturing PAGE containing 8 M urea.

### Primase assays

Unless otherwise stated, reactions (10 μl) containing 20 mM Tris–Ac (pH 7.9), 50 mM KAc, 10 mM Mg(Ac)_2_, 0.1 mg/ml BSA, 50 nM GP55 or its mutants, and 10 μM oligodeoxynucleotide templates or 10 nM ssM13 DNA (New England Biolabs), 100 μM ATP, GTP, UTP, and CTP, and 0.13–0.19 μM [α-^32^P]ATP (PerkinElmer) were incubated at 37°C for 15 min, and then were terminated by adding 5 μl of 95% formamide dye containing 20 mM EDTA and heated for 3 min at 75°C. The samples were analyzed by 25% denaturing PAGE containing 3 M urea.

### *De novo* DNA replication assays

Reactions (10 μl) containing 20 mM Tris–Ac (pH 7.9), 50 mM KAc, 10 mM Mg(Ac)_2_, 0.1 mg/ml BSA, 50 nM GP55 or its mutants, 10 nM ssM13 DNA (New England Biolabs), and the indicated NTPs and dNTPs were incubated at 37°C for the indicated time. For native agarose gel electrophoresis, 1 μl of protease K (Beyotime, China) was added to the reaction mixture, followed by incubation at 50°C for 15 min to digest proteins. Then, 2.2 μl of 6× loading dye containing 60 mM EDTA was added prior to electrophoresis. For alkaline agarose gel electrophoresis, protease K digestion was omitted. The reaction was directly terminated by adding 2 μl of 6× loading dye containing 60 mM EDTA before electrophoresis.

### Helicase assays

Heli-42nt-F (TAGATAGCCATAGATAGCATGCTAGTCACTGTTCGAGCACCA) was annealed to 5′-FAM-labeled Heli-42nt-R (TGGTGCTCGAACAGTGACTAGCTACGATAGATACCGATAGAT) to construct the substrate for helicase assays. Unless otherwise stated, assays were performed in a 10 μl reaction volume containing 800 nM GP55exo-, 50 nM substrate (Heli-42/42 nt), 1 μM non-labeled Heli-42nt-R, and indicated 4 mM NTP (A, U, C, or G) or dNTP (dA, dT, dC, dG, or dU) in reaction buffers (20 mM Tris–Ac, 50 mM KAc, 8 mM Mg(Ac)_2_, pH 7.9). The reaction mixtures were incubated at 37°C for 30 min. After incubation, the reaction mixtures were mixed with 2 μl of 6× Gel Loading Dye containing 0.48% SDS (New England Biolabs) and examined by 10% nondenaturing polyacrylamide gel in 1× TBE.

### Alkaline agarose gel analysis of the primer extension products

A 15-nt primer (CCCAGTCACGACG*T*T, where * indicates phosphorothioate modification) was labeled at 5′ end using T4 Polynucleotide Kinase (New England Biolabs) and [γ-^32^P]ATP (PerkinElmer), and then annealed to ssM13 DNA. Reactions (10 μl) containing 20 mM Tris–Ac (pH 7.9), 50 mM KAc, 10 mM Mg(Ac)_2_, 0.1 mg/ml BSA, 500 μM dNTPs, 9 nM primed ssM13 DNA, and 100 nM GP55 or ΔN(1–439) were incubated at 37°C for the indicated time, and then terminated by adding 2 μl of 6× loading dye. The samples were analyzed on a 0.8% alkaline agarose gel. T4 (without strand displacement activity) and Phi29 (with strand displacement activity) DNA polymerases were used as negative and positive controls, respectively.

### GP55 structure prediction

The structure of GP55 was predicted by AlphfaFold2 [15]. The best ranking structure was collected for further analysis, and visualized using PyMOL. Proteins were structurally aligned using PyMOL or DALI server [16] (http://ekhidna2.biocenter.helsinki.fi/dali/).

### Phylogenetic analysis

The amino acid sequence of GP55 was used as a query in a PSI-BLAST search against the NCBI non-redundant (nr) database, identifying over 1000 GP55 homologs (Supplementary Table S2). Multiple sequence alignment and phylogenetic tree construction were conducted using the online tool Clustal Omega, available at the European Bioinformatics Institute (EBI) website (https://www.ebi.ac.uk/Tools/msa/clustalo/). Default parameters were applied, and the results of both the multiple sequence alignment and phylogenetic tree were downloaded. The generated phylogenetic tree was visualized using the Interactive Tree of Life (iTOL) v5 [17], an online tool accessible at https://itol.embl.de.

## Results

### GP55 represents a group of multi-domain replicases predominantly found in human-associated bacteriophages

**Figure 1.**
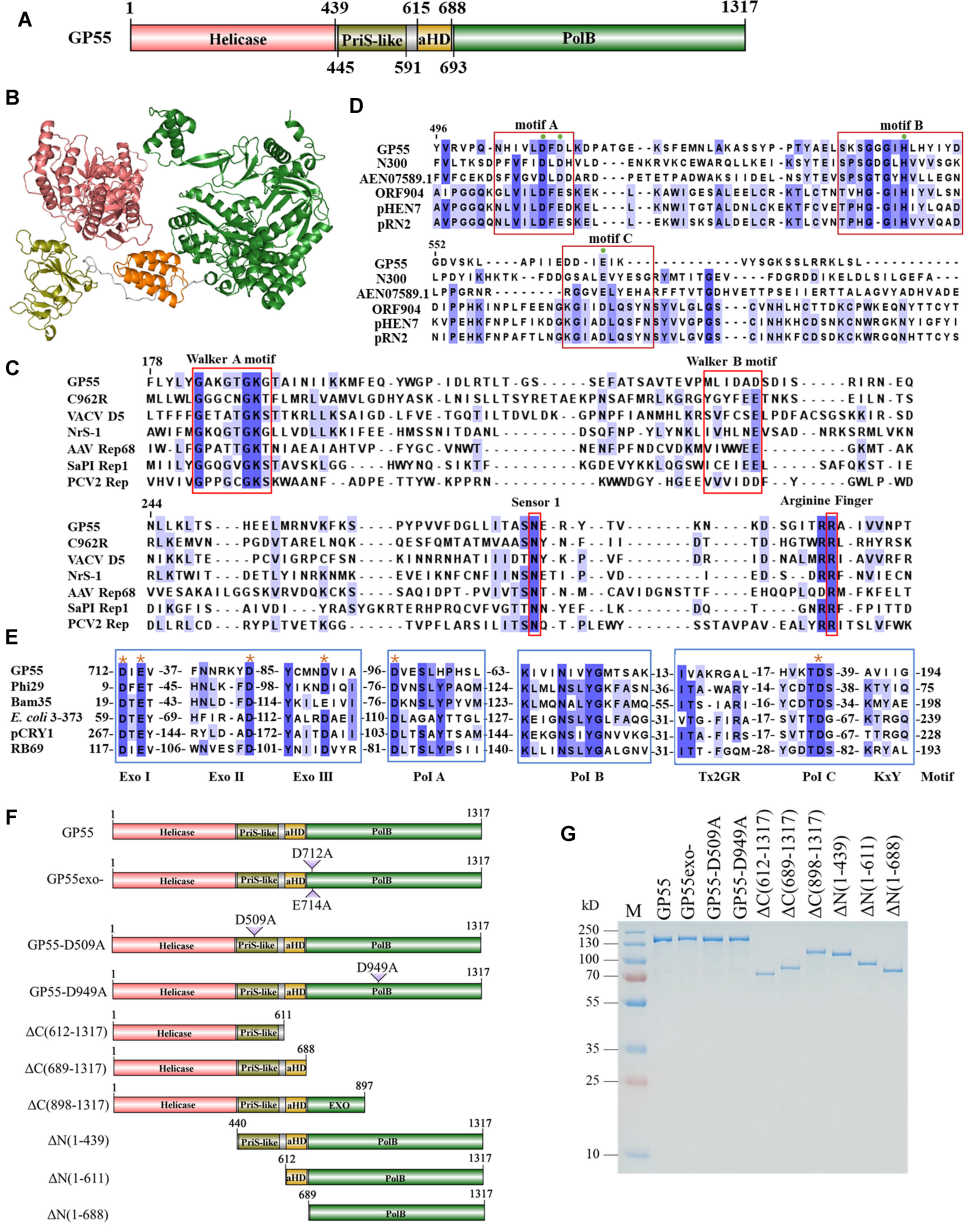
Sequence analysis showing that GP55 is a large multi-domain replicase. (**A**) The domain architecture of GP55. (**B**) Predicted structural model of GP55 generated using AlphaFold2. (**C**) Multiple sequence alignment of the GP55 helicase domain with those of other characterized helicase domain-containing proteins, based on [23]. (**D**) Multiple sequence alignment of the GP55 PriS-like domain with other PrimPol proteins, based on [7]. (**E**) Multiple sequence alignment of the GP55 PolB domain with other characterized PolB DNA polymerases, based on [5]. (**F**) Schematic illustration of the constructed GP55 mutants. (**G**) SDS–PAGE analysis of the purified GP55 and its mutants.

As the most abundant and diverse organisms in the world, viruses may replicate their genomes in unconventional ways through unique replicases. In this study, we noticed that ORF55 of lactococcal phage 1706 encodes a large replication-associated protein, GP55, which comprises 1317 amino acid residues [13]. GP55 exhibits a domain organization distinct from all known replicases. It comprises four domains: a helicase domain, a PriS-like domain, an unknown α-helix domain (aHD), and a family PolB domain (Fig. 1A). The predicated GP55 structure also reveals three relatively independent domains (Fig. 1B). Multiple sequence alignment showed that the helicase domain contains conserved Walker A motif, Walker B motif, sensor 1 residue, and arginine finger (Fig. 1C); the PriS-like domain contains conserved motifs A, B, and C found in AEP (Fig. 1D); the PolB domain contains conserved motifs in the exonuclease subdomain and polymerase subdomain similar to those in phi29 DNA polymerase [18–20] (Fig. 1E). Unlike AEP proteins, which contain not only a catalytic domain, such as PriS domain, but also a large accessory subunit (PriL) or helix bundle subdomain (HBD) essential for primase activity [21], GP55 lacks this subdomain. Although it contains an aHD following the PriS-like domain, the sequence of the aHD domain shows no homology to the HBD. Based on the sequence alignment results, we identified essential residues in PriS-like domain, exonuclease subdomain, and polymerase subdomain. We further constructed, expressed, and purified key residue-inactivating mutants and domain-deletion mutants of GP55 (Fig. 1F and G).

Furthermore, sequence homology searches identified over 1000 GP55 homologs in the currently available NCBI non-redundant (nr) database (Supplementary Fig. S1). Phylogenetic analysis revealed that GP55 homologs are encoded by phages infecting diverse bacteria phyla (Supplementary Fig. S1). Interestingly, these host bacteria predominantly belong to human-associated bacteria (Supplementary Fig. S1), such as those found in the human gut or saliva.

### GP55 exhibits DNA polymerase, 3′–5′ exonuclease, and helicase activities

**Figure 2.**
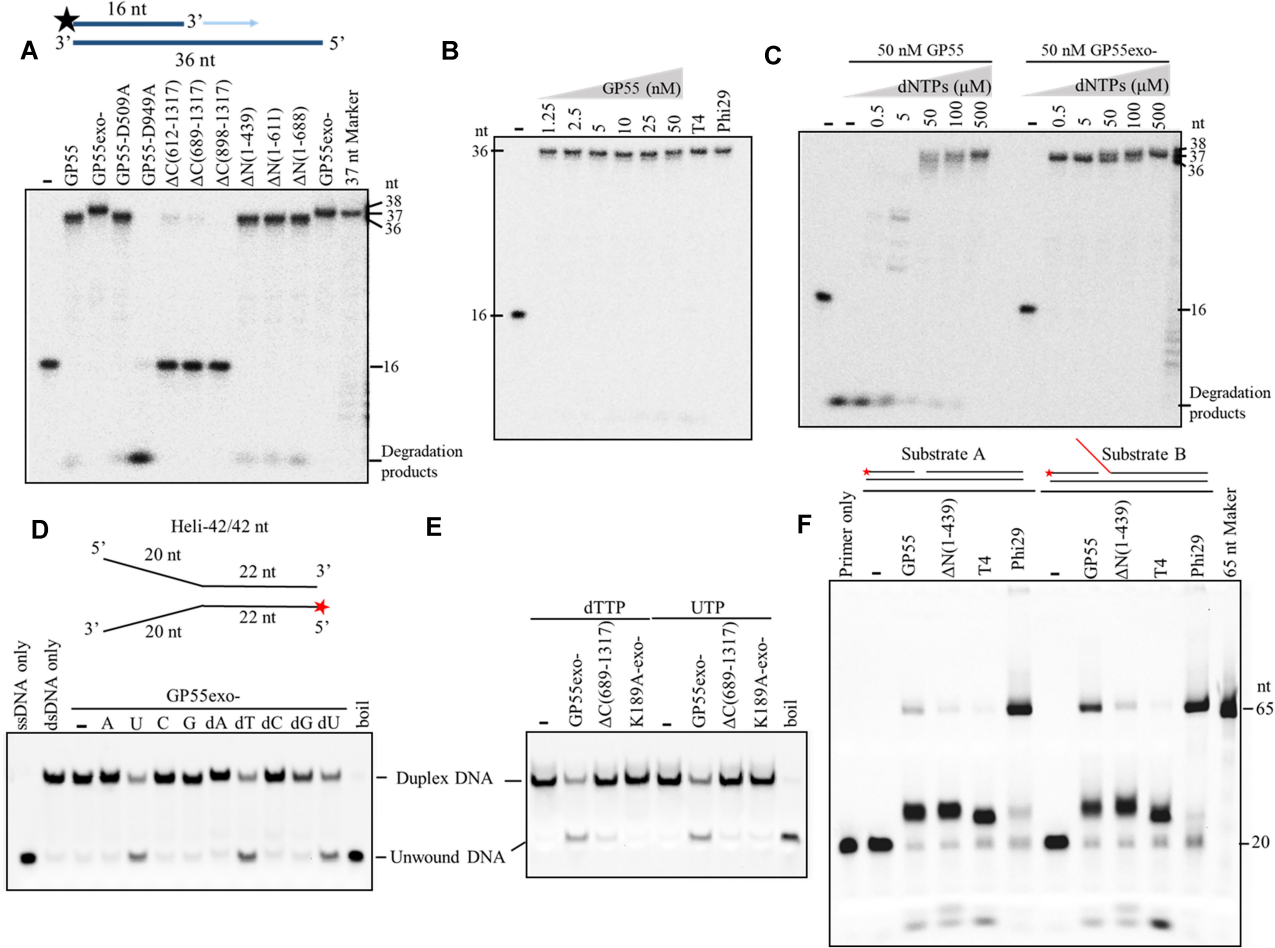
The DNA polymerase activity, 3′–5′ exonuclease activity, and helicase activity of GP55. (**A**) Extension of ^32^P-labeled 16/36-mer primer/template duplex substrate by GP55 and its mutants. (**B**) Primer extension by various amounts of GP55. (**C**) Primer extension by GP55 and GP55exo- in the presence of different concentrations of dNTPs. Samples in panels (A)–(C) were examined by 10% denaturing PAGE containing 7 M urea. (**D**) DNA unwinding assays catalyzed by GP55 in the presence of different kinds of NTPs or dNTPs. (**E**) Comparative analysis of DNA unwinding activity between GP55 and its mutants [ΔC(689–1317) and K189A-exo-]. Reaction mixtures were terminated by adding 6× Gel Loading Dye containing 0.48% SDS (New England Biolabs) in panels (D) and (E). (**F**) Strand displacement activity of GP55 examined using two different DNA substrates.

As GP55 contains a typical PolB domain with conserved motifs similar to those in phi29 DNA polymerase, we first examined its DNA polymerase activity. A 5′-^32^P labeled 16/36-mer primer/template duplex substrate was used to assess the DNA polymerase activities of GP55 and its mutants (Fig. 2A). The results showed that GP55 synthesizes expected 36-nt extension product, confirming its DNA polymerase activity (Fig. 2A). As anticipated, deletion of the PolB domain or mutation of key residues in the polymerase subdomain abolished DNA polymerase activity, while deletion of other domains (but not the PolB domain) retains the activity, demonstrating that the PolB domain is solely responsible for the GP55’s DNA polymerase activity (Fig. 2A). We also observed that polymerase-deficient mutant (GP55-D949A) exhibits exonuclease activity in the primer extension assays (Fig. 2A). Further analysis revealed that GP55 exhibits 3′–5′ exonuclease activity on both single-stranded DNA (ssDNA) and dsDNA (Supplementary Fig. S2). As expected, the putative exonuclease subdomain is essential for this activity. Deletion of the exonuclease subdomain or inactivation of its key residues (D712A and E714A) resulted in complete loss of the exonuclease activity (Supplementary Fig. S3A). Interestingly, the full-length GP55 shows a higher exonuclease activity compared to helicase domain deletion mutants (Supplementary Fig. S3B). That may be because the helicase domain facilitates DNA binding during cleavage. We also found that the exonuclease-deficient mutant (GP55exo-) adds a single nucleotide to the 3′ end of the extension product (Fig. 2A), a behavior similar to other exonuclease-deficient DNA polymerases such as Taq DNA polymerase. Primer extension assays further demonstrated efficient primer elongation, even at concentrations as low as 1.25 nM, highlighting GP55’s proficiency as a DNA polymerase (Fig. 2B). Subsequent assays with varying dNTPs concentrations revealed the competition between the primer extension activity and exonuclease activity of GP55. Equilibrium is influenced by dNTP concentrations (Fig. 2C). GP55exo- is more active in extension activity and adds extra base at 3′ end at high dNTP concentrations (Fig. 2C). We further examined the nucleotide misincorporation during primer extension by GP55 or GP55exo-. GP55 exhibits only exonuclease activity when provided with a single type of dNTP, while GP55exo- incorporates correct nucleotide at low concentrations and misincorporated incorrect nucleotides at high concentrations. These results indicate the proofreading potential of GP55 as a faithful DNA polymerase (Supplementary Fig. S4).

Multiple sequence alignment revealed that GP55 contains a helicase domain (Fig. 1C). To investigate whether GP55 exhibits helicase activity, DNA unwinding assays were performed using GP55exo- (to avoid degradation of the short DNA substrates by its exonuclease activity) mutant in the presence of various NTPs or dNTPs. Interestingly, the results showed that GP55exo- displays DNA unwinding activity exclusively in the presence of UTP or dTTP (Fig. 2D). We further investigated the effects of UTP and dTTP concentrations on the helicase activity (Supplementary Fig. S5). The results showed that 4 mM dTTP and 6 mM UTP were optimal for the helicase activity of GP55. To further confirm the helicase activity, we constructed, expressed, and purified helicase domain-inactivated mutant K189A-exo- (Supplementary Fig. S6), and compared the helicase activity of GP55exo- with those of ΔC(689–1317) and K189-exo-. As expected, K189A-exo- lost the helicase activity (Fig. 2E). Interestingly, the DNA polymerase domain-deletion mutant (ΔC(689–1317)) exhibited only minimal helicase activity, indicating that the DNA polymerase domain of GP55 affects the helicase function. Since the GP55 helicase domain belongs to the SF3 helicase family, we wondered whether it forms a hexameric ring structure, similar to other SF3 helicase domains [22, 23]. Interestingly, SEC–MALS analysis revealed that GP55 mainly forms a dimer, whereas ΔN(1–439) exists as a monomer (Supplementary Fig. S7 A and B), indicating that the helicase domain mediates the dimerization of GP55.

Given that GP55 harbors helicase activity, we next investigated whether it also possesses strand displacement activity. As shown in Fig. 2F, we used two synthetic DNA substrates to evaluate the strand displacement activity of GP55. The results showed that GP55 indeed possesses strand displacement activity, which is largely attributable to its helicase activity, as removal of the helicase domain markedly diminished this function.

### *De novo* DNA replication by the primase and DNA polymerase activities of GP55

**Figure 3.**
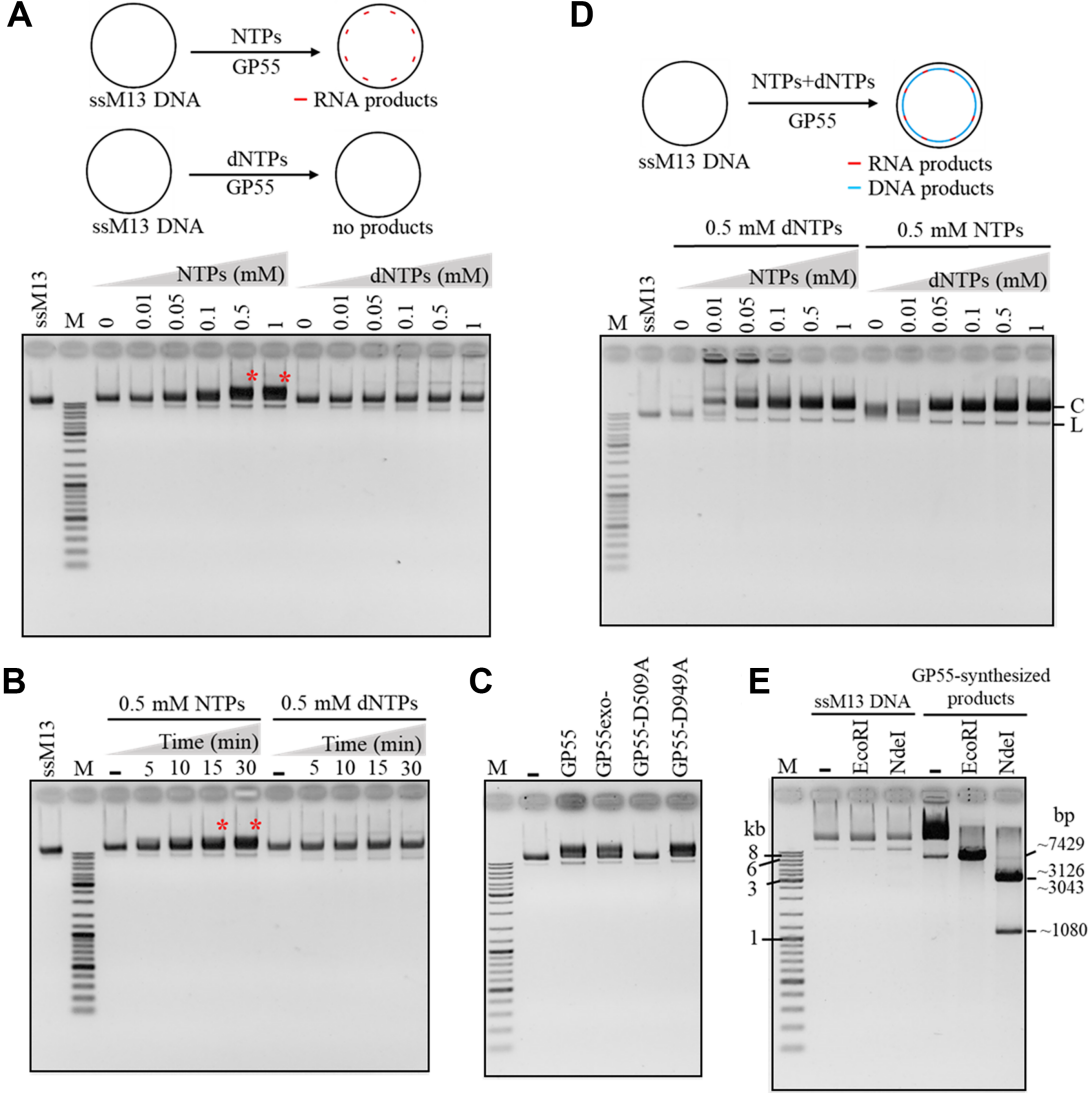
RNA primer synthesis and DNA *de novo* synthesis by GP55. (**A**) Oligonucleotide synthesis on ssM13 DNA by GP55 in the presence of various concentrations of NTPs or dNTPs. Prominent bands were indicated by stars. (**B**) Time course of oligonucleotide synthesis by GP55 in the presence of 0.5 mM NTPs or 0.5 mM dNTPs. Prominent bands were indicated by stars. (**C**) Comparison of the RNA synthesis by GP55 and its mutants (GP55exo-, GP55-D509A, and GP55-D949A). The reactions were performed at 37°C for 15 min in the presence of 0.5 mM NTPs. (**D**) DNA *de novo* synthesis on ssM13 DNA by GP55 in the presence of various combinations of NTPs and dNTPs. C: circular M13 dsDNA; L: linear M13 dsDNA. The purchased circular ssM13 DNA contains a small amount of linear ssM13 DNA, which was replicated by GP55 to generate linear M13 dsDNA. (**E**) GP55-replicated dsDNA products confirmed by cleavage of EcoRI (New England Biolabs) or NdeI (New England Biolabs). The M13 DNA contains one EcoRI cleavage site (5′-GAATTC-3′) and three NdeI cleavage sites (5′-CATATG-3′). M13 dsDNA was synthesized by GP55 in the presence of 10 nM ssM13 DNA, 0.5 mM NTPs, and 0.5 mM dNTPs. The ssM13 DNA and the synthesized dsDNA products were digested with 10 U of EcoRI and NdeI, respectively.

As GP55 is a replicase and contains a PriS-like domain, we hypothesized that GP55 possesses primase activity, capable of synthesizing oligonucleotide primers, despite lacking the putative PriL or HBD, which are essential for primer synthesis in typical AEP proteins. To test this, we examined its oligonucleotide primer synthesis activity using ssM13 DNA. We first examined primer synthesis under varying concentrations of NTPs or dNTPs. Significant products were detected at higher concentrations of NTPs (0.5–1 mM), indicating RNA primer synthesis (Fig. 3A). The amounts of synthesized products increased progressively with reaction time (Fig. 3B). In contrast, no significant products were observed in reactions containing dNTPs (Fig. 3A and B). To identify the domain of GP55 responsible for RNA primer synthesis, we compared the RNA primer synthesis activities of GP55 and its key residue mutants (Fig. 3C). The results confirmed that the PriS-like domain is crucial for RNA primer synthesis.

Notably, the presence of both NTPs and dNTPs resulted in more robust and significant product synthesis (Fig. 3D), suggesting that the RNA primer synthesized by GP55 could be further extended into longer DNA products. These findings not only confirm GP55’s RNA primer synthesis ability but also highlight its effective coordination between primase and DNA polymerase activities during DNA replication (Fig. 3D). Interestingly, we observed the synthesis of much larger DNA products when using 0.5 mM dNTPs with low concentrations of NTPs (Fig. 3D). We speculate that at higher NTP concentrations, GP55 may synthesize longer RNA primers and initiate replication at more sites along the ssM13 DNA. Since GP55 preferentially unwinds duplex regions with 5′ overhangs (Fig. 2F), an increased number of RNA primer–DNA template hybrids may hinder unwinding, thereby reducing its strand displacement activity. We subsequently confirmed the GP55-synthesized products using restriction endonucleases EcoRI and NdeI. As expected, the synthesized products were cleaved by EcoRI and NdeI, whereas the original ssM13 DNA remained intact (Fig. 3E). The sizes of the cleavage fragments matched the theoretical fragment sizes, validating the complete replication of ssM13 DNA by GP55 in the presence of both dNTPs and NTPs (Fig. 3E).

### Characterization of the primase activity of GP55

Since GP55 can synthesize RNA primers, we next sought to identify its primase recognition site. By screening short DNA oligonucleotides (including those derived from ssM13 DNA), combined with systematic truncation and mutagenesis, we identified a strong primase recognition sequence 5′-CCAAC-3′, at which the GP55 efficiently catalyzes the synthesis of RNA primers (Supplementary Fig. S8). However, it is worth noting that although GP55 requires sequence recognition for primer synthesis, its sequence specificity is less stringent (Supplementary Fig. S8) than that observed for other AEP family proteins [21].

**Figure 4.**
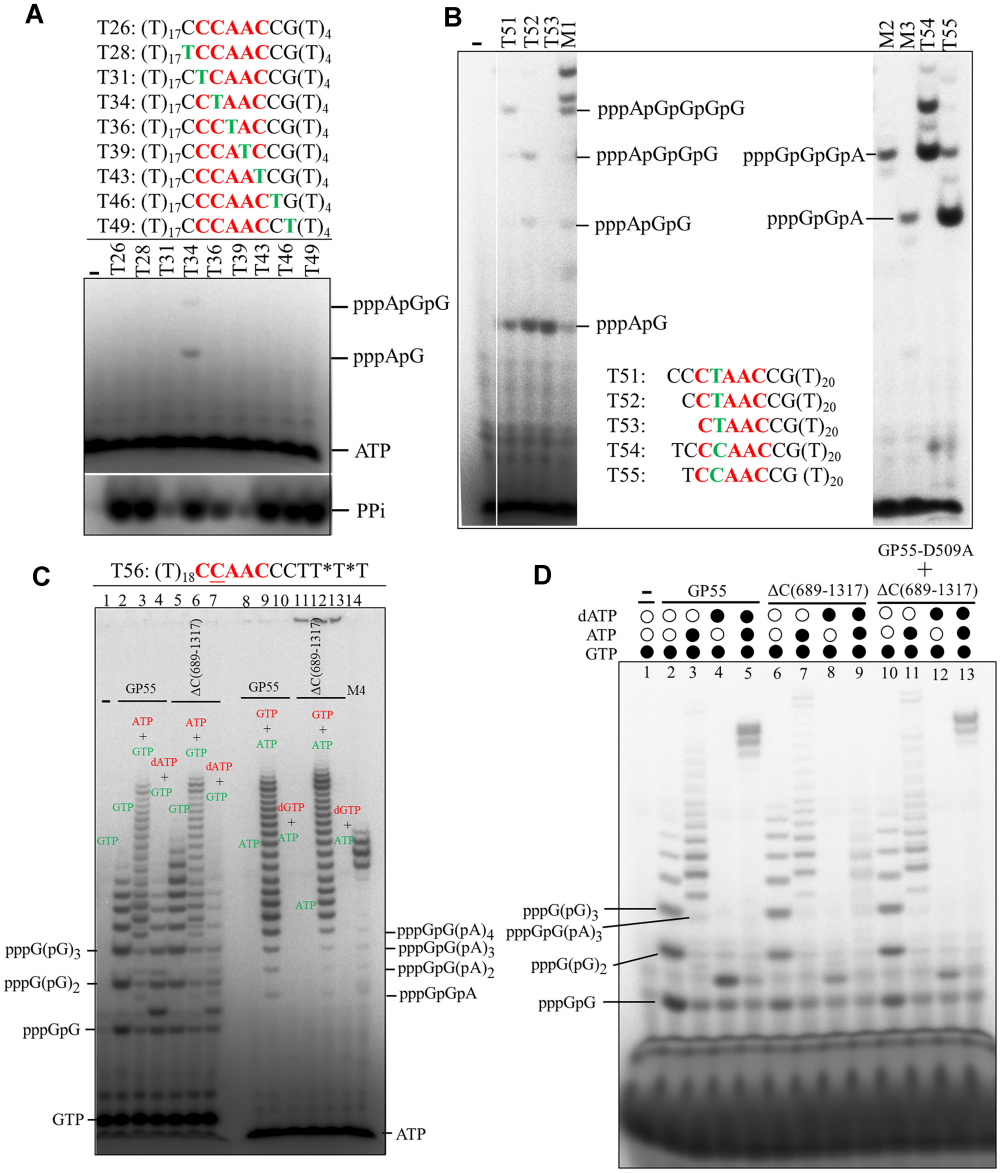
Dissection of the primer synthesis by GP55. (**A**) Identification of the initiation site of primer synthesis using [γ-^32^P]ATP labeling. RNA primers were synthesized by GP55 on various templates in the presence of 100 μM GTP, UTP, CTP, and ∼0.4 μM [γ-^32^P]ATP. The released inorganic pyrophosphate (PPi) indicated RNA primer synthesized by GP55. (**B**) Confirmation of the initiation site using [α-^32^P]ATP labeling. RNA primers were synthesized by GP55 on the templates T51–T55 in the presence of 100 μM GTP, UTP, and CTP, and 0.19 μM [α-^32^P]ATP. Marker M1, M2, and M3 were synthesized by N300 domain of NrSPol as described previously [7]. (**C**) Analysis of primer synthesis on the template T56. The oligonucleotide primers were synthesized by 50 nM GP55 or ΔC(689–1317) in the presence of different combinations of nucleotides (ATP, dATP, GTP, and dGTP) at 100 μM. Lanes 1–7 contain samples labeled with [α-^32^P]GTP, while lanes 8–14 contain samples labeled with [α-^32^P]ATP. Marker M4 was synthesized by N300 domain of NrSPol as described previously [7]. The * indicates phosphorothioate modification at the 3′ terminus of T56. (**D**) RNA primer synthesis coupled with DNA synthesis by GP55. RNA primer or DNA products were synthesized on template T56 by GP55, ΔC(689–1317), or the combined action of GP55-D509A and ΔC(689–1317), in the presence or absence of 100 μM ATP or 500 μM dATP.

With this preferred recognition sequence, we aimed to identify the exact initiation site of primer synthesis. On template T26 containing the strong primase recognition motif (5′-CCAAC-3′), we systematically introduced single thymidine (T) substitutions at positions within and adjacent to the recognition site to generate templates T28, T31, T34, T39, T43, T46, and T49 (Fig. 4A). The introduced T substitutions in DNA templates allow ATP to initiate primer synthesis. We used [γ-^32^P]ATP in the reactions so that any labeled RNA or oligonucleotides must begin with ATP. As shown in Fig. 4A, the production of inorganic pyrophosphate (PPi) confirmed RNA primer synthesis on these templates. However, [γ-^32^P]-labeled di- and trinucleotide products were only observed with template T34, in which the second cytosine (C) of the 5′-CCAAC-3′motif (counting from the 5′ end) was substituted by a T base (Fig. 4A). No labeled products were detected from templates with T substitutions at other positions within or adjacent to the 5′-CCAAC-3′ motif (Fig. 4A). These results suggest that primer synthesis is initiated at the second base from the 5′ end of the recognition site.

To further confirm the precise initiation site, we examined primer synthesis on templates T51–T55 using [α-^32^P]ATP to label the RNA products. As shown in Fig. 4B, the detection of di-, tri-, and tetranucleotides with templates T51–T53 indicated that primer synthesis initiates at the T base within the primase recognition site 5′-CTAAC-3′. Similarly, templates T54 and T55, which contain the strong primase recognition site 5′-CCAAC-3′, produced the expected tri- and tetranucleotides, supporting that primer synthesis initiates at the second base of the 5′-CCAAC-3′ (with the underlined base indicating the primer initiation site). The yield of RNA from 5′-CCAAC-3′ is much higher than that from 5′-CTAAC-3′ (comparing T54 and T55 to T51–T53 in Fig. 4B), suggesting that GTP is preferred than ATP to initiate primer synthesis by GP55.

We further characterize the nucleotide preference of GP55 primase using template T56 containing a primase recognition site 5′-CCAAC-3′. The truncated GP55 mutant ΔC(689–1317) retained primase activity comparable to full-length GP55, both synthesized poly(G) primers when only GTP was provided (Fig. 4C, lanes 2 and 5). On the shortened templates TG2, TG3, and TG4, we found that GP55 produced oligonucleotides longer than the distance (2–4 nucleotides) from the initiation site to the 5′ end of the template (Supplementary Fig. S9), suggesting that template slippage occurred during primer synthesis. However, these poly(G) products, which did not perfectly match the templates, failed to be extended upon the addition of dATP (comparing lanes 2 and 4 in Fig. 4C). In contrast, when ATP was provided together with GTP, GP55 robustly synthesized oligoribonucleotides ranging from 2 to 22 nucleotides in length, with the longest products reaching the end of the DNA template (Fig. 4C, lanes 3, 6, 9, and 12), whereas no such activity was observed with dATP (Fig. 4C, lanes 4 and 7). These results suggest that GP55 preferentially synthesizes RNA primer, in contrast to the mixed r/dNTPs usage observed in human PrimPol [24].

Interestingly, in reactions containing GTP, ATP, and dATP, GP55 [but not the polymerase-deficient ΔC(689–1317)] predominantly generated extension products that nearly reached the template end, suggesting that the RNA primers were efficiently extended by the polymerase domain (Fig. 4D). When the primase-deficient GP55-D509A mutant and the polymerase-deficient ΔC(689–1317) mutant were combined, similar extension products were observed (Fig. 4D), suggesting that GP55’s primase and polymerase domains can cooperate *in trans*.

Additionally, we examined whether the synthesized poly(G) could serve as primers for extension on additional templates (T58–T61) containing varying numbers of C bases, which can base pair with the poly(G) primer. Primer extension was observed only on template T61, which contains four consecutive C bases, indicating that poly(G) primers require a minimum of 4-nt base pairing to support extension (Supplementary Fig. S10).

In summary, we found that GP55 primase exhibits limited sequence specificity, with 5′-CCAAC-3′ identified as a strong priming site. GTP is preferred to initiate the primer synthesis at the second C base within the site. The primase domain synthesizes RNA primers ranging from 2 to 22 nucleotides in length without deoxynucleotide incorporation, and a primer as short as 4 nucleotides is sufficient to support extension by the polymerase domain.

### Primer synthesis by GP55 requires aHD

**Figure 5.**
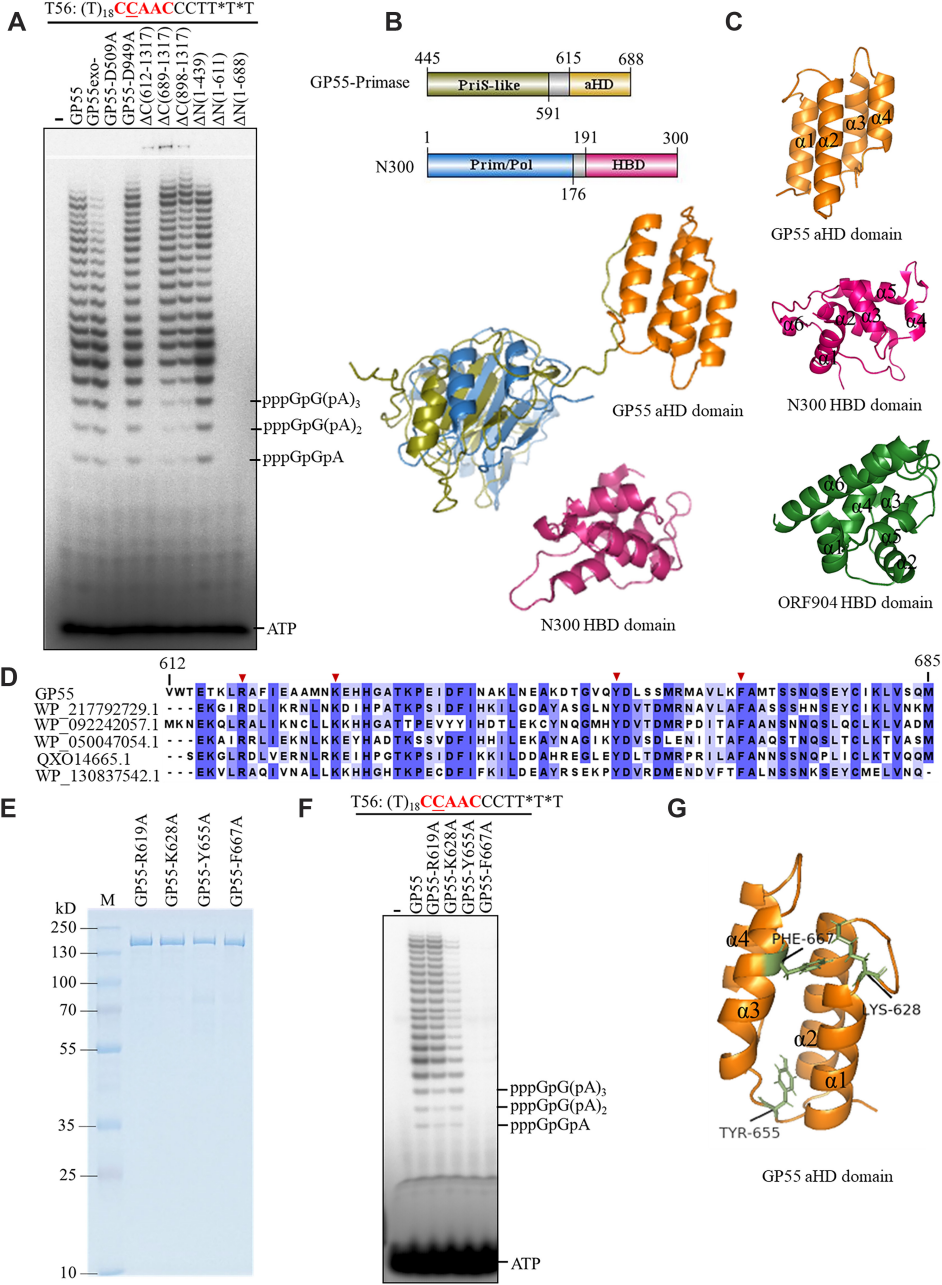
Characterization of the aHD of GP55. (**A**) Comparison of the RNA primer synthesis activity of GP55 and its mutants on template T56. (**B**) Structural superposition of GP55 primase domain with the truncated mutant (N300) of NrSPol, a PrimPol protein (PDB: 6A9W). (**C**) Structural comparison of the GP55 aHD, the N300 HBD, and ORF904 HBD (PDB: 3M1M). (**D**) Sequence alignment of the GP55 aHD and its homologous sequences. Arrowheads indicated the conserved residues selected for functional analysis. (**E**) SDS–PAGE analysis of the purified GP55 and its aHD-inactivated mutants. (**F**) Comparison of the primer synthesis activity of GP55 and its aHD-inactivated mutants. (**G**) Structural mapping of the key residues identified in aHD.

GP55 contains an unknown function domain, aHD (Fig. 1A). To elucidate its role, we investigated the primer synthesis activity of various GP55 mutants. The results revealed that both the PriS-like domain and the adjacent aHD are essential for RNA primer synthesis (Fig. 5A), and the GP55 primase domain encompasses the two domains. The aHD appears functionally analogous to the HBD of PrimPol proteins, as both domains are composed of α-helices. However, structural alignments showed no homology between the two domains (Fig. 5B and C). Specifically, the aHD is composed of two pairs of parallel α-helices, whereas the HBD consists of six interwoven α-helices (Fig. 5C). A structural comparison of aHD with the PDB database using DALI [16] further confirmed that the aHD domain shares no homology with known primase family proteins. Thus, aHD represents a new functional domain responsible for primase activity. We further aligned the aHD sequence of GP55 with homologous proteins and identified several conversed residues (Fig. 5D). Based on this alignment, we selected four residues for mutational analysis and constructed corresponding mutants (Fig. 5E). Activity assays revealed that three of these mutations significantly reduced primer synthesis activity. Notably, mutations at Y655 and F667 completely abolish GP55’s primer synthesis activity (Fig. 5F). Structural analysis indicated that these critical residues are located on the α-helices of aHD or within loops connecting the helices (Fig. 5G). These results underscore the essential role of aHD in primer synthesis.

### Characterization of the *de novo* DNA synthesis activity of GP55

**Figure 6.**
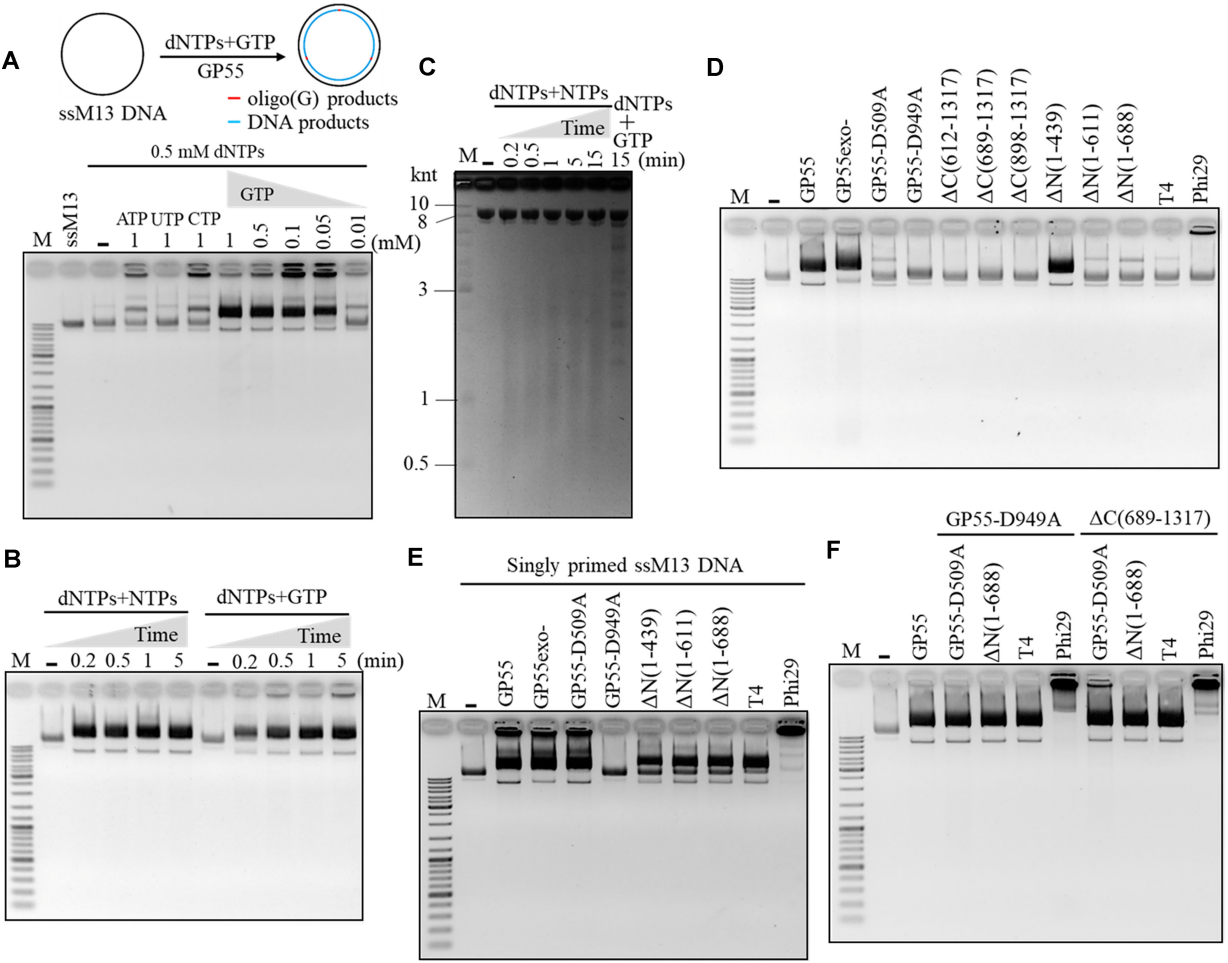
Characterization of DNA replication by GP55 and comparison with commercial DNA polymerases. (**A**) *De novo* DNA replication on ssM13 DNA template by GP55 in the presence of dNTPs and a single type of NTP. (**B**) Time course analysis of DNA replication on the ssM13 DNA by GP55 in the presence of dNTPs and either all four NTPs or GTP alone. (**C**) Alkaline (denaturing) agarose gel analysis of the DNA products synthesized by GP55 at different time points. (**D**) Comparison of the *de novo* DNA synthesis of GP55, its mutants, and commercial DNA polymerases (T4 and Phi29) on ssM13 DNA template. Reactions contained 10 nM ssM13 DNA, 500 μM dNTPs and 500 μM NTPs, and 50 nM GP55 or its mutant, 3 U T4 (New England Biolabs) or 10 U Phi29 (New England Biolabs). (**E**) DNA extension activities on singly primed ssM13 DNA by GP55, its mutants, and commercial DNA polymerases (T4 and Phi29). Reactions contained 10 nM ssM13 DNA, 200 nM primer M2 (5′-CCCAGTCACGACG*T*T-3′), 500 μM dNTPs and 500 μM NTPs, 50 nM GP55 or its mutant, 3 U T4, or 10 U Phi29. (**F**) Examination of RNA primers synthesized by GP55 mutants [GP55-D949A and ΔC(689–1317)] and their extension by GP55, its mutants, and commercial DNA polymerases (T4 and Phi29).

We next conducted a more detailed investigation of GP55’s enzymatic characteristics. It has been shown that GP55 efficiently replicates ssDNA in the presence of NTPs and dNTPs (Fig. 3D). To further explore the *de novo* DNA synthesis activity of GP55, we examined DNA replication by GP55 on ssM13 DNA in the presence of all four dNTPs and a single type of NTP. Interestingly, a significant amount of DNA was synthesized in the presence of all four dNTPs and only GTP, and the replication products increased at higher GTP concentrations (Fig. 6A). However, only a minimal amount of replication products was produced when using all four dNTPs and either ATP or CTP (Fig. 6A). Almost no replication product was detected in the presence of all four dNTPs and only UTP (Fig. 6A). We further assessed the efficiency of DNA replication by GP55 on ssM13 DNA. The results demonstrated that ssM13 DNA could be efficiently replicated by GP55 in the presence of all four dNTPs and NTPs (Fig. 6B). In fact, 10 nM ssM13 DNA (7249 nt) was fully replicated by 50 nM GP55 in 0.2 min when using all four dNTPs and NTPs, whereas slightly more time was required in the presence of only GTP and all four dNTPs (Fig. 6B). Alkaline agarose gel electrophoresis analysis revealed smeared bands, indicative of newly synthesized DNA products, ranging in length from ∼600 to ∼4000 nt (Fig. 6C). However, when GTP was used as the sole NTP, distinct bands rather than a smear pattern were observed (Fig. 6C), suggesting less frequent initiation events under these conditions. Sequence analysis of the ssM13 genome revealed seven 5′-CCAAC-3′ motifs (four of which are 5′-CCCAAC-3′), known as strong primase recognition sites for GP55. Our results have shown that GP55 synthesizes poly(G) in the presence of GTP alone on the templates containing 5′-CCCAAC-3′ motif (Fig. 4C). The poly(G) primer can hybridize with templates containing 5′-CCCC-3′, enabling primer extension by GP55 (Supplementary Fig. S10). The ssM13 genome contains 11 motifs of 5′-CCCC-3′ including 2 5′-CCCCC-3′ motifs, likely allowing poly(G) primers to be extended on ssM13 template.

Next, we examined whether the key residue mutants, domain-truncated mutants, or the commercial DNA polymerase (T4 and Phi29) could replicate ssM13 DNA *de novo*. As expected, mutants lacking an active PolB domain or a functional primase domain of GP55 did not exhibit *de novo* synthesis activity (Fig. 6D). In contrast, deletion of the helicase domain did not affect *de novo* synthesis activity (Fig. 6D). Neither T4 nor Phi29 exhibited *de novo* synthesis activity (Fig. 6D). We further tested these enzymes on primed ssM13 DNA. As expected, T4 and Phi29 efficiently extended primers, with Phi29 exhibiting strand displacement activity (Fig. 6E). GP55 and its mutants containing an active PolB domain also exhibited primer extension activity (Fig. 6E). DNA products larger than the template were synthesized by GP55 but not the helicase domain-deletion mutants (Fig. 6E and Supplementary Fig. S11), further demonstrating that GP55 possesses strand displacement activity, which relies on its helicase domain. Moreover, the primer extension activity of helicase domain-deletion mutants was weaker than that of wild-type GP55 (Fig. 6E), indicating that the helicase domain also contributes to the efficiency of polymerase function.

To further characterize the *de novo* DNA synthesis activity of GP55, we tested primers synthesized by GP55 mutants that retained primase activity but lacked DNA polymerase activity [GP55-D949A and ΔC(689–1317)]. These primers were used as substrates to evaluate their extension by GP55 mutants deficient in primase activity [GP55-D509A and ΔN(1–688)] or by commercial DNA polymerase (T4 and Phi29). The results demonstrated that primers synthesized by GP55-D949A or ΔC(689–1317) could be efficiently extended by both GP55 mutants [GP55-D509A and ΔN(1–688)] and commercial DNA polymerase (T4 and Phi29) (Fig. 6F).

## Discussion

Common DNA replication relies on primases to synthesize short oligonucleotide primers that are then extended by DNA polymerases, as DNA polymerases cannot initiate DNA synthesis *de novo*. Typically, primases and DNA polymerases are distinct proteins. However, our study reveals a novel class of replicases, exemplified by GP55, which harbors both primase and DNA polymerase in a single protein. GP55, unlike previously reported polymerases with *de novo* DNA synthesis activities [2–6], relies on two active centers responsible for RNA primer synthesis and DNA extension, respectively. Previous research indicated conflicts between primase activity and DNA polymerase activity within a single protein with the same catalytic domain [7]. However, GP55, with separate primase and DNA polymerase catalytic centers, exhibits robust primase and DNA polymerase activities.

GP55 is comprised of an N-terminal helicase domain, two central domains including the PriS-like domain and the newly identified aHD, and a C-terminal PolB domain, collectively exhibiting helicase, primase, 3′–5′ exonuclease, and DNA polymerase activities. The coexistence of these four replication-related enzymatic activities within a single protein is unprecedented. One key question is how GP55 maintains the high efficiency of all four activities simultaneously. Notably, these domains are not merely fused together but functionally interconnected. For example, deletion of the polymerase domain of GP55 will dramatically affect its helicase activity (Fig. 2E).

We have shown that the PriS-like domain and aHD are responsible for primer synthesis, defining the primase function of GP55 (Fig. 5A). The PriS-like domain is homologous to the catalytic domain of NrSPol [7]. However, unlike NrSPol and other AEP primases [21], GP55 lacks essential auxiliary protein domains, such as PriL or HBD. Instead, the newly identified aHD domain appears to substitute for the role of the HBD. Notably, aHD is not homologous to HBD and has no equivalent among characterized primase proteins, indicating that aHD represents a novel evolutionary adaptation. This adaptation may have been driven by the need for compatibility between primase and PolB domains. By employing aHD as the bridging auxiliary domain, GP55 achieves robust primase activity while retaining efficient DNA polymerase with intrinsic 3′–5′ exonuclease activity. Additionally, we found that GP55 recognizes specific but less stringent DNA sequences compared to traditional AEP proteins such as NrSPol and ORF904, which use the 5′-GTG-3′ motif as a strong primase recognition site. The substitution of HBD with aHD as the essential auxiliary domain may partially account for this difference.

In this study, another interesting finding is that GP55 exhibits UTP/dTTP-dependent helicase activity, which distinguishes it from most helicase proteins that rely on ATP. While interestingly, the only other known helicase to rely on dTTP for DNA unwinding, to our knowledge, is the gp4 helicase from bacteriophage T7 [25]. We also discovered that the helicase domain enhances both the DNA polymerase activity (Fig. 6E) and exonuclease activity (Supplementary Fig. S3 B) and imparts strand displacement activity to GP55 (Fig. 6E). Interestingly, SEC–MALS analysis revealed that GP55 forms a dimer (Supplementary Fig. S7), suggesting that it may function as a dimer during DNA replication. Our results demonstrated that the primase and polymerase activities of GP55 can function *in trans* (Fig. 4D), suggesting that one subunit within the dimer may be responsible for primer synthesis, while the other subunit carries out primer extension. These findings indicate a model in which GP55 functions as a self-contained two-subunit replisome with coordinated enzymatic activities. One open question is whether this mode of replication offers a more streamlined and efficient mechanism. Additionally, we have observed that GP55 exhibits strand displacement activity and can synthesize ultra-long DNA products, similar to Phi29. However, to fully develop the practical potential of GP55, conditions for the balance of the multiple functions by GP55 need further optimization and exploration, particularly regarding its fidelity, processivity, and strand displacement activity.

Interestingly, proteins homologous to GP55 are abundant in human-associated bacteriophages listed in the NCBI non-redundant (nr) database, although there could be sampling bias due to the over-representation of human metagenome sequences in the current databases. These bacteriophages are known to play important roles in regulating gut microbiota homeostasis and may influence disease development both directly and indirectly [26, 27]. However, our understanding of these phages remains limited. In this study, our findings provide new insights into the genome replication of these human-associated bacteriophages containing GP55 homologs.

## Supplementary Material

gkaf542_Supplemental_Files

## Data Availability

The data underlying this article are available in the article and in its online supplementary material.
