# Supplementary material for: A four-in-one replicase integrating key enzymatic activities for DNA replication
Source: Nucleic Acids Res. 2025 Jun 23;53(12):gkaf542. doi: 10.1093/nar/gkaf542 (PMC12205980; doi:10.1093/nar/gkaf542)
Supplement: gkaf542_Supplemental_Files [file gkaf542_supplemental_files.zip › 20250522-GP55 Supplementary information.pdf]

Supplementary information

A Four-in-One Replicase Integrating Key Enzymatic Activities For DNA Replication

Yuxin Zhang[1], Xueling Lu[1], Bin Zhu[1,2]*, and Fengtao Huang[1]*

[1]Key Laboratory of Molecular Biophysics, the Ministry of Education, College of Life Science and Technology, Huazhong University of Science and Technology, Wuhan, Hubei 430074, China;

[2]Shenzhen Huazhong University of Science and Technology Research Institute, Shenzhen 518063, China

*To whom correspondence should be addressed. Email: bin_zhu@hust.edu.cn (contact for materials); huang_fengtao@126.com

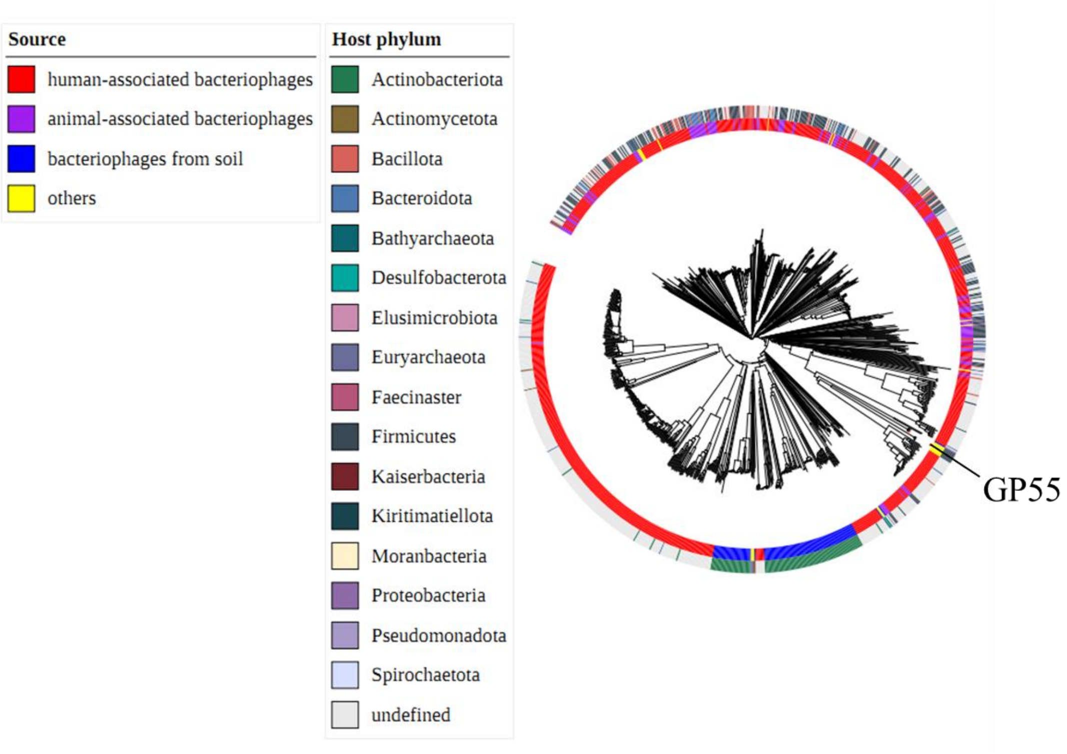

Figure S1. Phylogenetic tree showing GP55 homologs encoded by bacteriophages infecting diverse bacteria phyla.

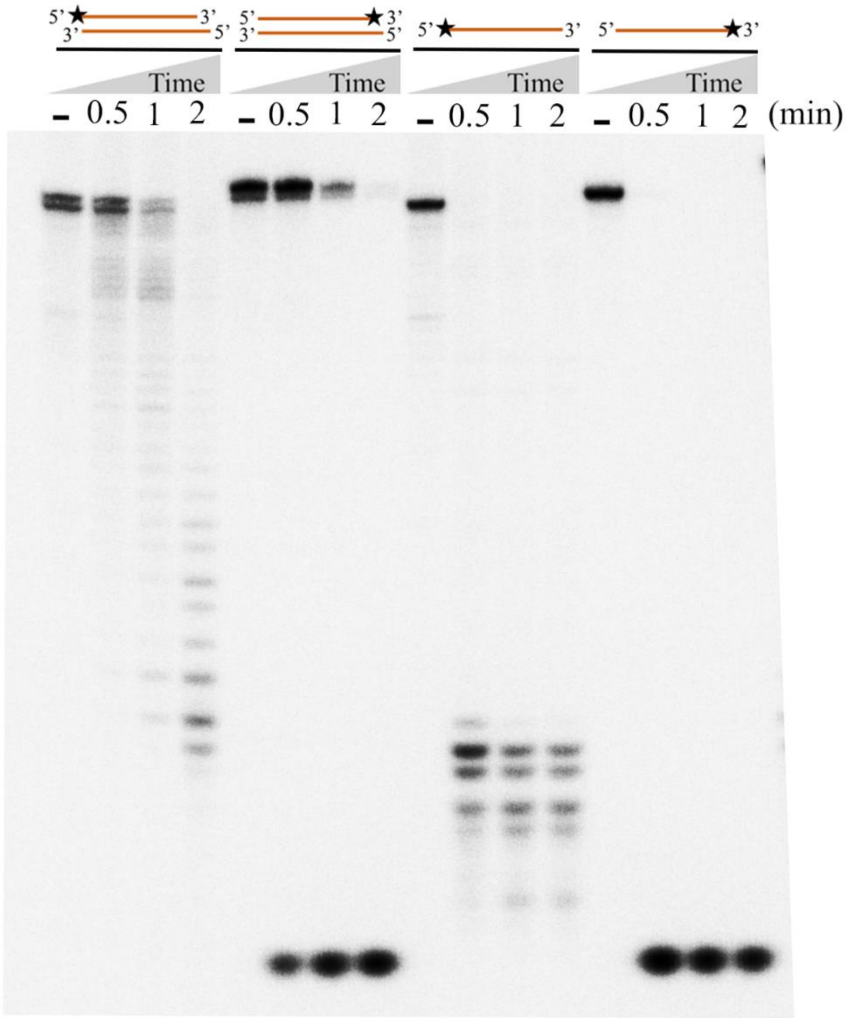

Figure S2. Orientation of GP55 exonuclease activity. A 36-nt oligonucleotide (5'-GAGATCCTATCGAGTAGCTCTGAAGACCCTGACATG-3') was radio-labeled at 5' or 3' end using T4 polynucleotide kinase (New England Biolabs) and [γ-^{32}P] ATP (Perkin Elmer) or terminal transferase (New England Biolabs) and [α-^{32}P] dGTP (Perkin Elmer), respectively. The labeled oligonucleotides were annealed to a 41-nt template (5'-CCCCCCATGTCAGGGTCTTCAGAGCTACTCGATAGGATCTC-3') to form corresponding 5' or 3' labeled duplexes. The labeled DNA was cleaved by GP55 in reaction buffer (20 mM Tris-Ac, 50 mM KAc, 10 mM $Mg(Ac)_2$, 0.1 mg/ml BSA, pH 7.9) at 37°C for indicated time. The reactions were terminated by adding 5 μl of 95% formamide dye containing 20 mM EDTA and heated for 3 min at 90°C. The samples were examined by 20% denaturing PAGE containing 8 M urea.

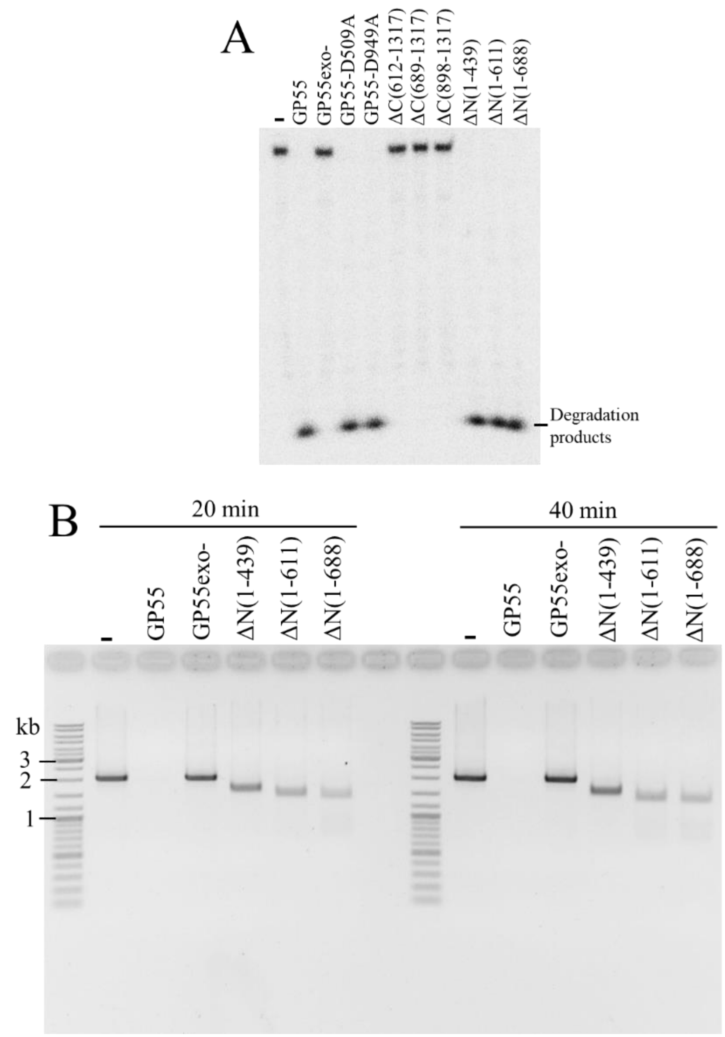

Figure S3. The exonuclease activities of GP55 and its mutants. (A) Investigation of the exonuclease activities of GP55 and its mutants toward the 36 bp DNA. (B) Comparison of the exonuclease activities of GP55 and the truncated mutants. 2 kb DNA fragments were cleaved by GP55 and its mutants for 20 min and 40 min, respectively. The results showed that helicase domain deletion affects the exonuclease activity of GP55.

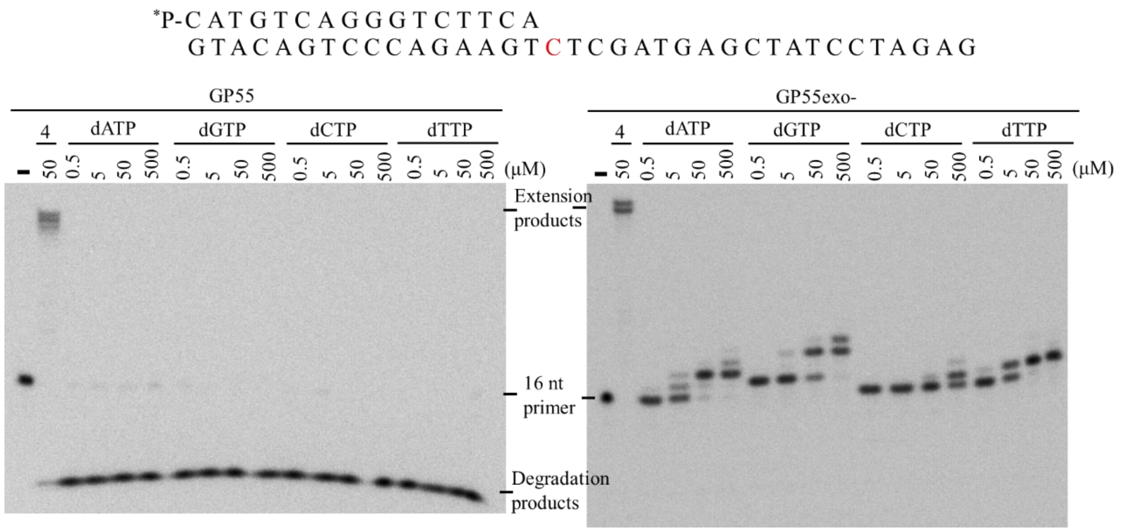

Figure S4. Nucleotide insertion preference by the exonuclease-deficient GP55 in the presence of increasing amounts of each dNTP.

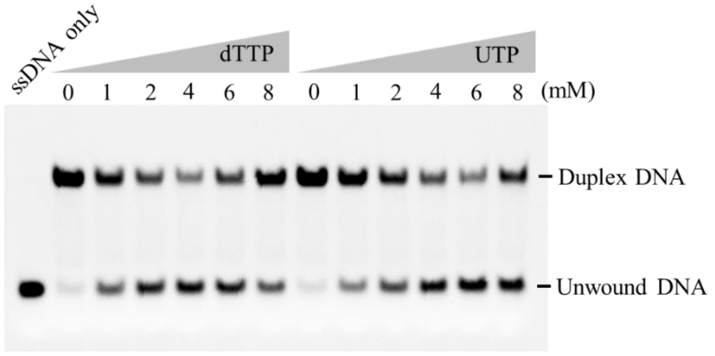

Figure S5. Effects of dTTP or UTP concentrations on the DNA unwinding activity of GP55. The reaction mixtures containing 50 nM substrate and 800 nM GP55exo-, and various concentrations of dTTP or UTP were incubated at 37°C for 30 min, and then were examined by 10% nondenaturing polyacrylamide gel.

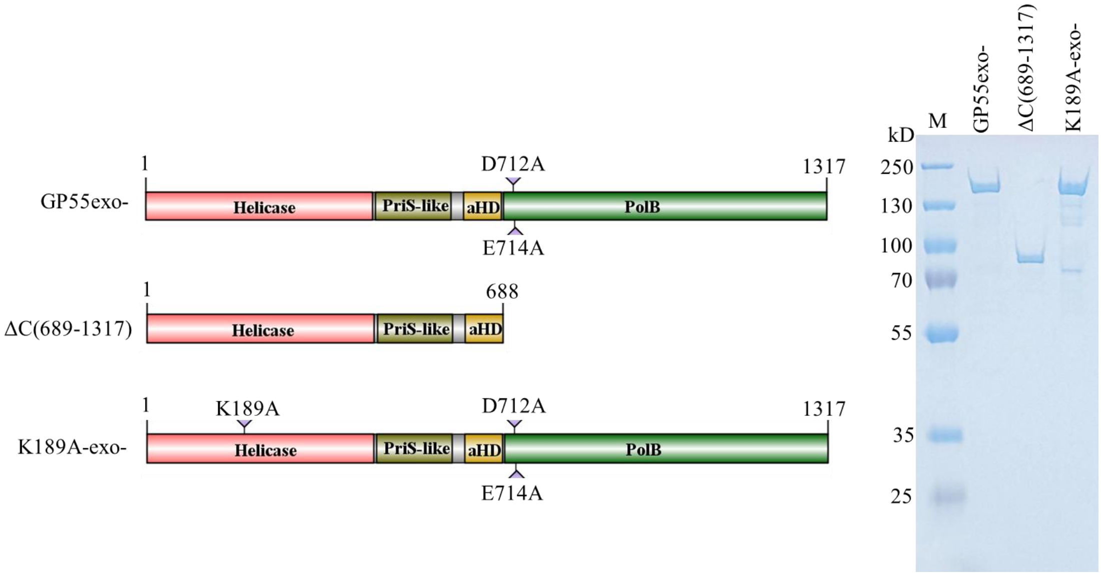

Figure S6. SDS-PAGE analysis of the purified GP55exo-, ΔC(689-1317) and K189A-exo-.

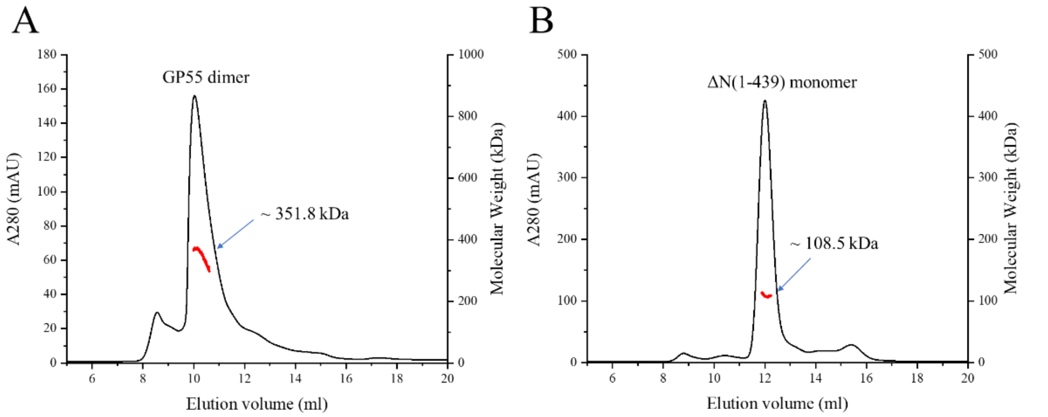

Figure S7. Size analysis of GP55 and its helicase domain-deletion mutant, ΔN(1-439). (A-B) SEC-MALS analysis of the molecular weights of GP55 (A) and ΔN(1-439) (B). The theoretical molecular weights of GP55 (including the his tag) and ΔN(1-439) (including the his tag) are 153.7 kDa and 102.8 kDa, respectively. The results showed that GP55 forms a dimer, while ΔN(1-439) exists as monomer.

A

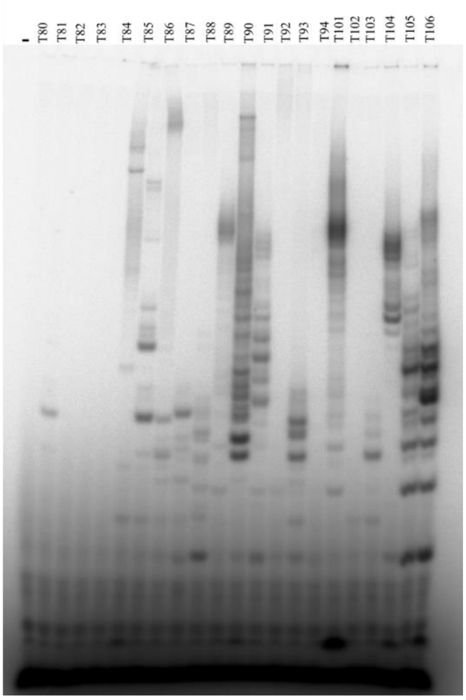

T80: CTAGGCACCGG
T81: CTAGGCACCG
T82: CTAGGCACC
T83: CTAGGCAC
T84: CGTTCTAGAACTATGTAGGTTTTGCAGAATTAGGCCTAGGCACCGGT
T85: CTAGGCACCGGTCGTTCTAGAACTATGTAGGTTTTGCAGAATTAGGC
T86: AATGCTACTACTATTAGTAGAATTGATGCCACCTTTTCAGCTCGCGCCCCAAATGAAAA
T87: AAATGAAAATATAGCTAAACAGGTTATTGACCATTTGCGAAATGTATCTAATGGTCAAA
T88: ATGGTCAAACTAAATCTACTCGTTCGCAGAATTGGGAATCAACTGTTACATGGAATGAA
T89: TGGAATGAAACTTCCAGACACCGTACTTTAGTTGCATATTTAAAACATGTTGAGCTACA
T90: TGAGCTACAGCACCAGATTCAGCAATTAAGCTCTAAGCCATCCGCAAAAATGACCTCTT
T91: TGACCTCTTATCAAAAGGAGCAATTAAAGGTACTCTCTAATCCTGACCTGTTGGAGTTT
T92: TTGGAGTTTGCTTCCGGTCTGGTTCGCTTTGAAGCTCGAATTAAAACGCGATATTTGAA
T93: ATATTTGAAGTCTTTCGGGCTTCCTCTTAATCTTTTTGATGCAATCCGCTTTGCTTCTG
T94: TTGCTTCTGACTATAATAGTCAGGGTAAAGACCTGATTTTTGATTTATGGTCATTCTCG
T101: TGGAATGAAACTTCCAGACACCGTACTTTAtttttttt
T102: AGACACCGTACTTTAGTTGCATATTTAAAAaaaaa
T103: AGTTGCATATTTAAAACATGTTGAGCTACAtttttttt
T104: TGACCTCTTATCAAAAGGAGCAATTAAAGGtttttttt
T105: AGGAGCAATTAAAGGTACTCTCTAATCCTGtttttttt
T106: TACTCTCTAATCCTGACCTGTTGGAGTTTtttttttt

B

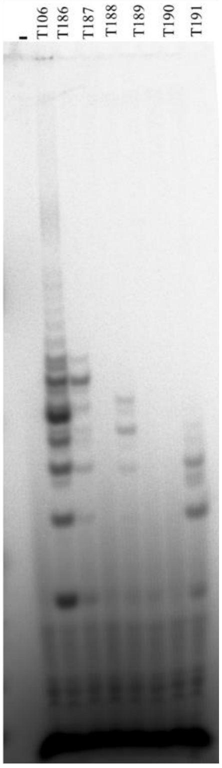

T106: TACTCTCTAATCCTGACCTGTTGGAGTTTtttttttt
T186: (T)$_5$**TACTCTCTAATCCTG**(T)$_5$
T187: (T)$_{15}$**TACTCTCT**(T)$_3$
T188: (T)$_{15}$**TCTCTAAT**(T)$_3$
T189: (T)$_{16}$**TAATCCT**(T)$_3$
T190: (T)$_{16}$**TCCTG**(T)$_4$
T191: (T)$_{11}$**CTAATCCTG**(T)$_5$

C

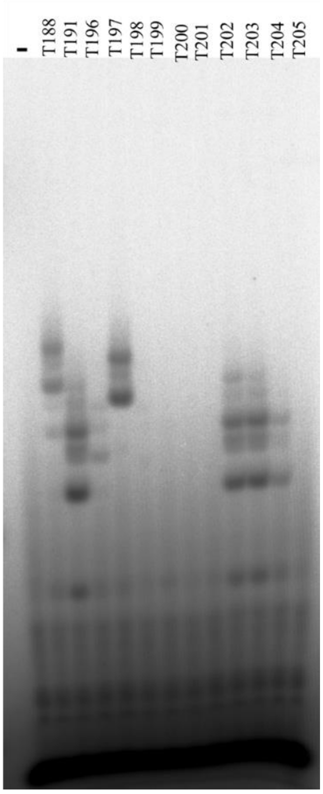

T188: TTTTTTTTTTTTTTTTTCTCTAATTTT
T196: TTTTTTTTTTTTTTTTTGTCTAATTTT
T197: TTTTTTTTTTTTTTTTTCACTAATTTT
T198: TTTTTTTTTTTTTTTTTCTGTAATTTT
T199: TTTTTTTTTTTTTTTTTCTCAAATTTT
T200: TTTTTTTTTTTTTTTTTCTCTTATTTT
T201: TTTTTTTTTTTTTTTTTCTCTATTTTT

T191: $(T)_{11}$**CTAATCCTG**$(T)_5$
T202: $(T)_{18}$**CTAATCCT**$(T)_3$
T203: $(T)_{18}$**CTAATC**$(T)_5$
T204: $(T)_{18}$**CTAAT**$(T)_6$
T205: $(T)_{18}$**GTCCT**$(T)_5$

D

T203: TTTTTTTTTTTTTTTTTTCTAATCTTTTT

T209: TTTTTTTTTTTTTTTTTACTAATCTTTTT
T210: TTTTTTTTTTTTTTTTTCCTAATCTTTTT
T211: TTTTTTTTTTTTTTTTTGCTAATCTTTTT
T212: TTTTTTTTTTTTTTTTTTATAATCTTTTT
T213: TTTTTTTTTTTTTTTTTTTTAATCTTTTT
T214: TTTTTTTTTTTTTTTTTTGTAATCTTTTT
T215: TTTTTTTTTTTTTTTTTTCAAATCTTTTT
T216: TTTTTTTTTTTTTTTTTTCCAATCTTTTT
T217: TTTTTTTTTTTTTTTTTTCGAATCTTTTT
T218: TTTTTTTTTTTTTTTTTTCTCATCTTTTT
T219: TTTTTTTTTTTTTTTTTTCTTATCTTTTT
T220: TTTTTTTTTTTTTTTTTTCTGATCTTTTT
T221: TTTTTTTTTTTTTTTTTTCTACTCTTTTT
T222: TTTTTTTTTTTTTTTTTTCTATTCTTTTT
T223: TTTTTTTTTTTTTTTTTTCTAGTCTTTTT
T224: TTTTTTTTTTTTTTTTTTCTAAACTTTTT
T225: TTTTTTTTTTTTTTTTTTCTAACCTTTTT
T226: TTTTTTTTTTTTTTTTTTCTAAGCTTTTT
T227: TTTTTTTTTTTTTTTTTTCTAATATTTTT
T228: TTTTTTTTTTTTTTTTTTCTAATTTTTTT
T229: TTTTTTTTTTTTTTTTTTCTAATGTTTTT
T230: TTTTTTTTTTTTTTTTTTCTAATCATTTT
T231: TTTTTTTTTTTTTTTTTTCTAATCCTTTT
T232: TTTTTTTTTTTTTTTTTTCTAATCGTTTT

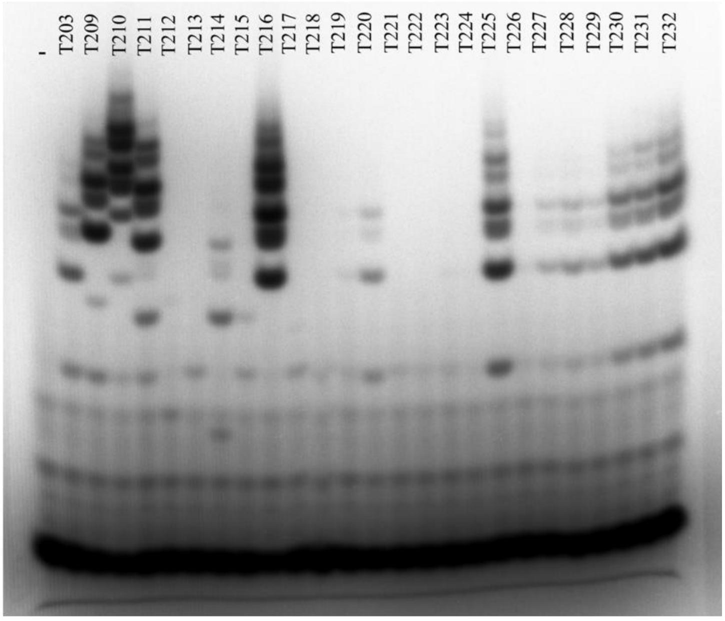

E

T216: (T)$_{17}$TCCAATCT(T)$_4$
T2: (T)$_{17}$ACCAATCT(T)$_4$
T3: (T)$_{17}$CCCAATCT(T)$_4$
T4: (T)$_{17}$GCCAATCT(T)$_4$
T5: (T)$_{17}$TACAATCT(T)$_4$
T6: (T)$_{17}$TTCAATCT(T)$_4$
T7: (T)$_{17}$TGCAATCT(T)$_4$
T8: (T)$_{17}$TCAAATCT(T)$_4$
T9: (T)$_{17}$TCTAATCT(T)$_4$
T10: (T)$_{17}$TCGAATCT(T)$_4$
T11: (T)$_{17}$TCCTATCT(T)$_4$
T12: (T)$_{17}$TCCCATCT(T)$_4$
T13: (T)$_{17}$TCCGATCT(T)$_4$
T14: (T)$_{17}$TCCATTCT(T)$_4$
T15: (T)$_{17}$TCCACTCT(T)$_4$
T16: (T)$_{17}$TCCAGTCT(T)$_4$
T17: (T)$_{17}$TCCAAACT(T)$_4$
T18: (T)$_{17}$TCCAACCT(T)$_4$
T19: (T)$_{17}$TCCAAGCT(T)$_4$
T20: (T)$_{17}$TCCAATAT(T)$_4$
T21: (T)$_{17}$TCCAATTT(T)$_4$
T22: (T)$_{17}$TCCAATGT(T)$_4$
T23: (T)$_{17}$TCCAATCA(T)$_4$
T24: (T)$_{17}$TCCAATCC(T)$_4$
T25: (T)$_{17}$TCCAATCG(T)$_4$

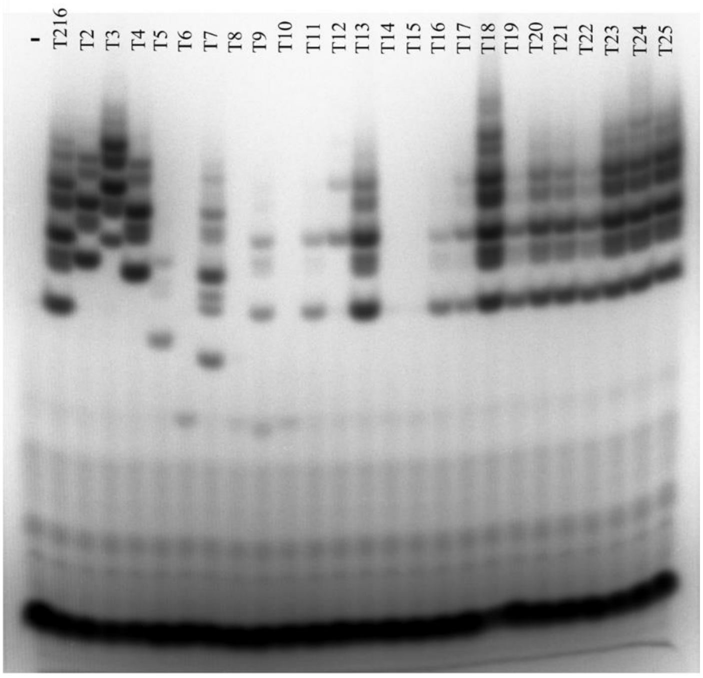

F

T216: (T)$_{17}$TCCAATCT(T)$_4$
T2: (T)$_{17}$ACCAATCT(T)$_4$
T3: (T)$_{17}$CCCAATCT(T)$_4$
T4: (T)$_{17}$GCCAATCT(T)$_4$
T5: (T)$_{17}$TACAATCT(T)$_4$
T6: (T)$_{17}$TTCAATCT(T)$_4$
T7: (T)$_{17}$TGCAATCT(T)$_4$
T8: (T)$_{17}$TCAAATCT(T)$_4$
T9: (T)$_{17}$TCTAATCT(T)$_4$
T10: (T)$_{17}$TCGAATCT(T)$_4$
T11: (T)$_{17}$TCCTATCT(T)$_4$
T12: (T)$_{17}$TCCCATCT(T)$_4$
T13: (T)$_{17}$TCCGATCT(T)$_4$
T14: (T)$_{17}$TCCATTCT(T)$_4$
T15: (T)$_{17}$TCCACTCT(T)$_4$
T16: (T)$_{17}$TCCAGTCT(T)$_4$
T17: (T)$_{17}$TCCAAACT(T)$_4$
T18: (T)$_{17}$TCCAACCT(T)$_4$
T19: (T)$_{17}$TCCAAGCT(T)$_4$
T20: (T)$_{17}$TCCAATAT(T)$_4$
T21: (T)$_{17}$TCCAATTT(T)$_4$
T22: (T)$_{17}$TCCAATGT(T)$_4$
T23: (T)$_{17}$TCCAATCA(T)$_4$
T24: (T)$_{17}$TCCAATCC(T)$_4$
T25: (T)$_{17}$TCCAATCG(T)$_4$

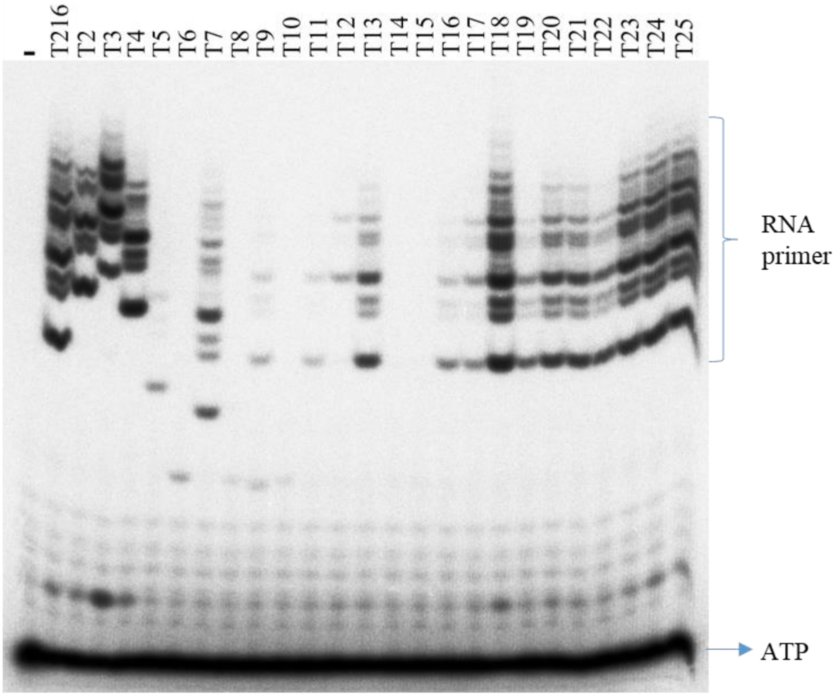

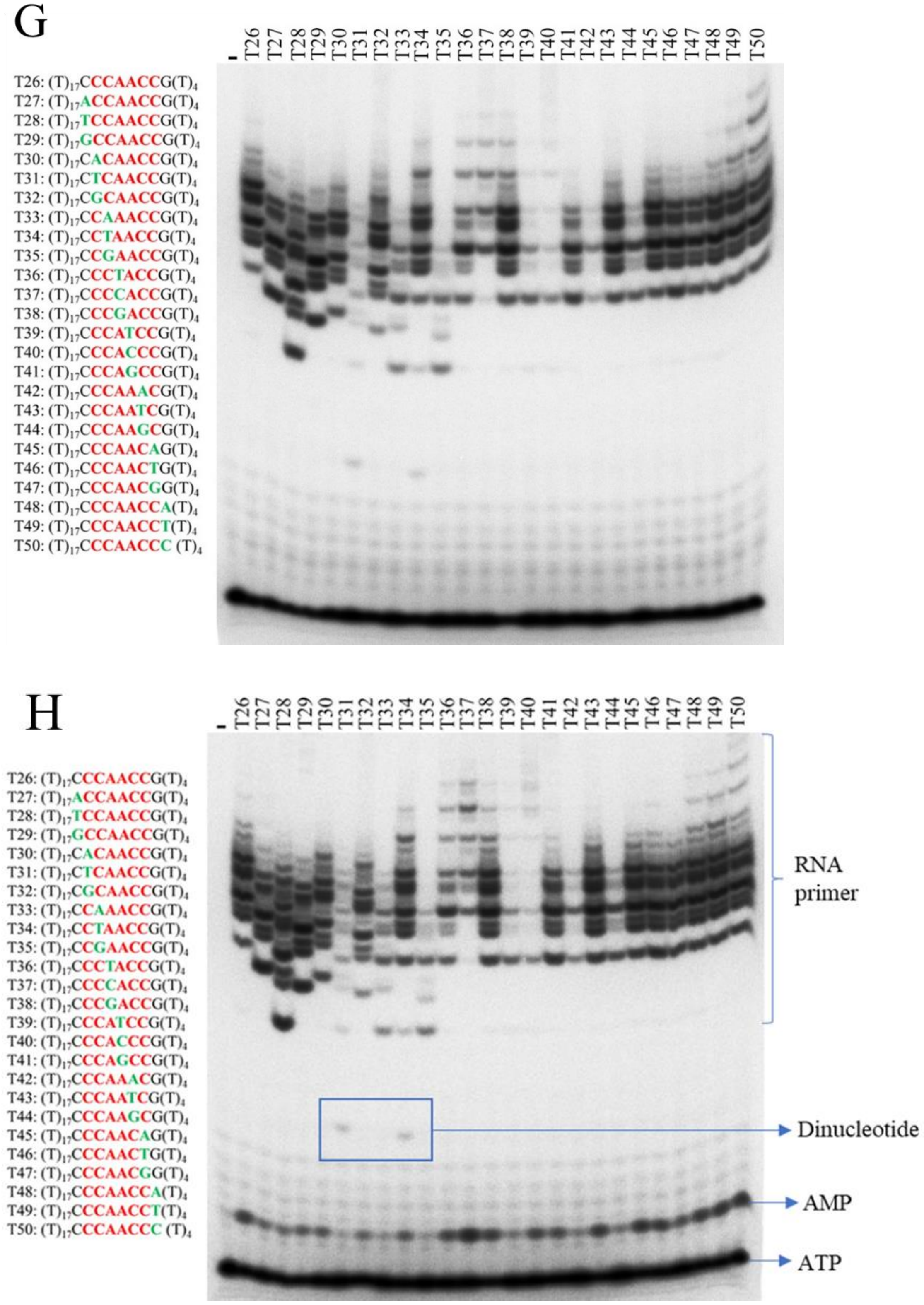

Figure S8. Identification of the primase recognition site of GP55. The 5'-CCAAC-3' motif as a strong primase recognition site was identified step by step. (A) A set of random templates were tested for their ability to support RNA primer synthesis by GP55. Among them, templates T86–T94 were derived from ssM13 DNA sequence, with T106 exhibiting the highest primase activity. **(B)** To narrow down the potential recognition motif within T106, a series of

truncated derivatives covering different regions of T106 were designed (with sequences highlighted in red derived from T106). Although the templates T188 and T191 showed reduced activity compared to T106, they retained detectable primase activity and contained much shorter core sequences, making them suitable for further motif identification. **(C)** Single-nucleotide substitutions were introduced into the core sequence of T188 to generate templates T196–T201, and truncation variants (T202–T205) of the T191 core sequence were designed. Among these, T203 exhibited comparable activity and contained a shorter core sequence, and was therefore selected as the starting point for further optimization. **(D)** Each base within the core sequence (5'-TCTAATCT-3') of T203 was systematically substituted with all three alternatives to generate Templates T209–T232. The RNA primer synthesis activity of these was evaluated, and T216 (core sequence: 5'-TCCAATCT-3') was identified as the most efficient template. **(E-F)** Based on T216, a second round of base substitutions was performed to generate Templates T2–T25, which were tested for primase activity using GP55exo- (E) and GP55 (F). The optimized template T26 (core sequence: 5'-CCCAACCG-3') was obtained by selecting the most favorable base at each position. **(G-H)** Template T26 and its core base-substituted mutants were designed based on the results shown in (E-F), and used to test the primase activity of GP55exo- (G) and GP55 (H). GP55 exhibited robust primase activity on template T26 and most of its variants, but showed sensitivity to substitutions within the 5'-CCAAC-3' motif, confirming this region as a strong primase recognition sequence.

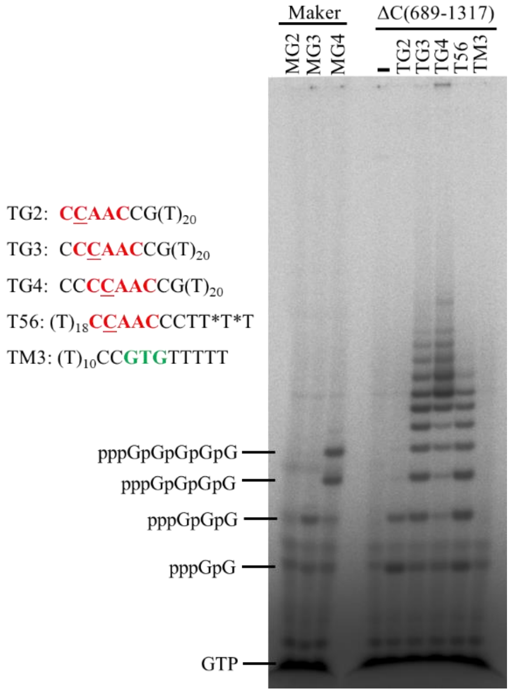

Figure S9. Poly(G) primer synthesis by ΔC(689-1317) on different templates. MG2, MG3 and MG4 as markers were synthesized by N300.

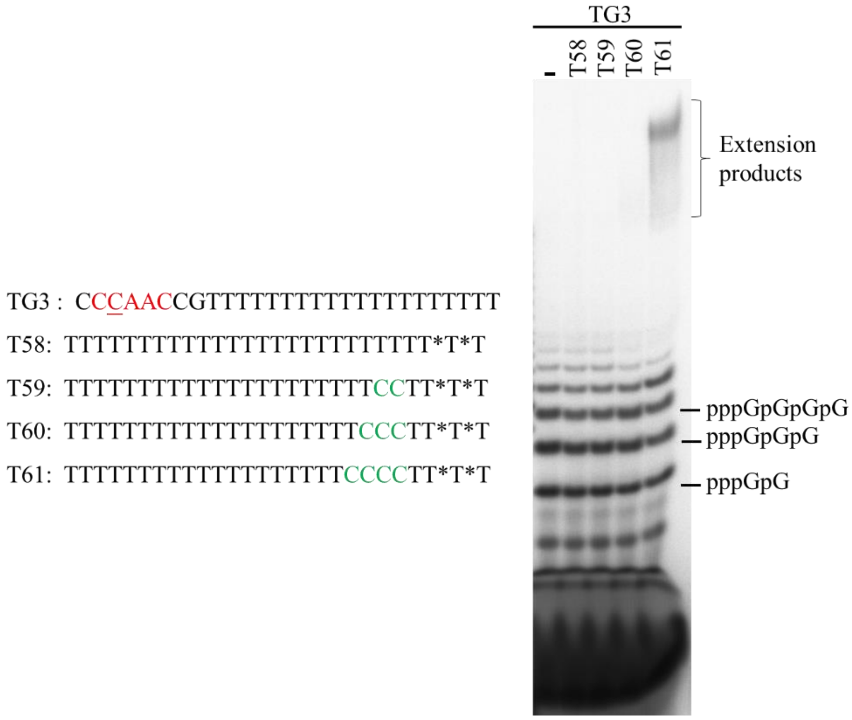

Figure S10. Examining extension products synthesized by GP55 in the presence of template TG3 and nucleotides (GTP and dATP), supplemented with one of the additional templates T58, T59, T60 or T61.

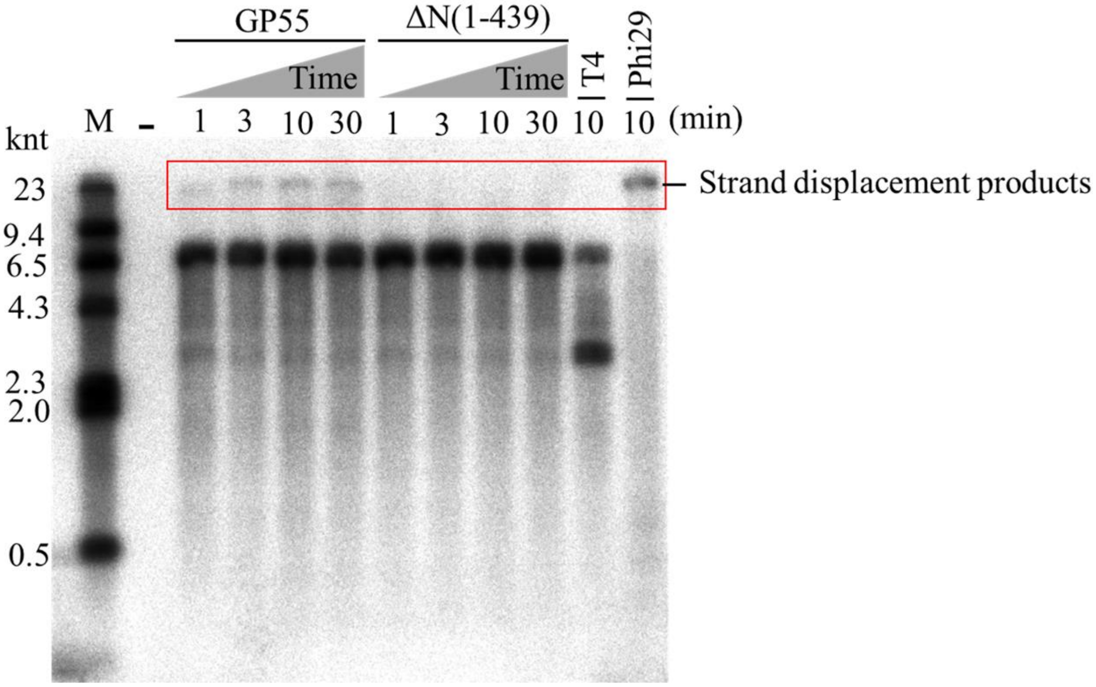

Figure S11. Comparison and analysis of the extension products synthesized by GP55 and ΔN(1-439). The assays were performed as described in *Materials and Methods*. The primer extension products on the ssM13 DNA template were analyzed on a 0.8% alkaline agarose gel.

Source Data

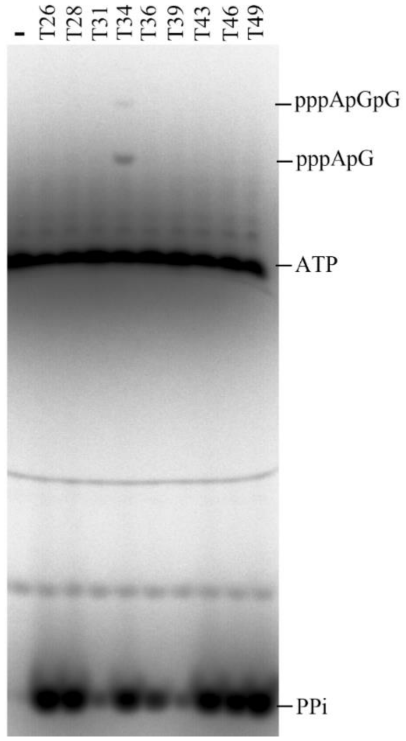

The uncropped figure related to Figure 4A.

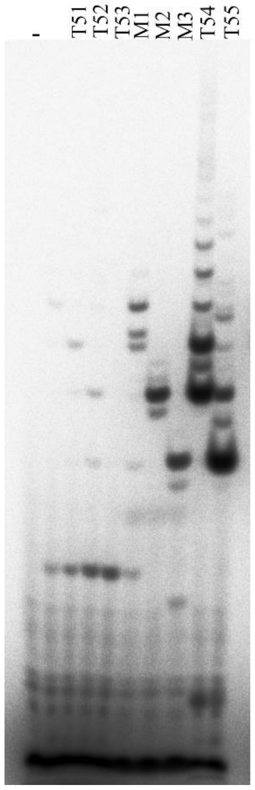

The uncropped figure related to Figure 4B.

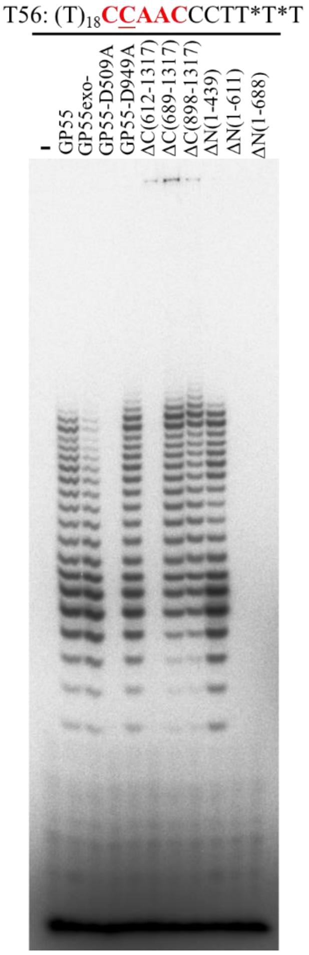

The uncropped figure related to Figure 5A.
