# Supplementary material for: A four-in-one replicase integrating key enzymatic activities for DNA replication
Source: Nucleic Acids Res. 2025 Jun 23;53(12):gkaf542. doi: 10.1093/nar/gkaf542 (PMC12205980; doi:10.1093/nar/gkaf542)
Supplement: gkaf542_Supplemental_Files [file gkaf542_supplemental_files.zip › GP55-Table S1.docx]

**Table S1. The codon-optimized nucleotide sequences of GP55 gene and the amino acid sequence of the full-length GP55.**

| **The codon-optimized nucleotide sequences of GP55 gene:**  ATGGACTTCTACAAGACCCTGGATGAACGTGTGACCAACCGTAACGCGAGCAGCGACTACGTTATTTGGCCGGATTTCAAATATGCGGGTGCGAAGGACATCGTGGTTAAAGGTGGCGATTTTTACGCGTATTGGAACGGCGAGACCTGGATTACCGACATCGATAGCCTGATTAAGGCGATCGACCAGGATAGCTGGGAACGTTTTAACGAGCTGCGTGCGCTGGACCCGAACCTGCGTATTAACGTGAAACAAATGGATTTCGCGAGCAGCGGTATCATGGCGAAGTTTCAGCAATACTGCCAGCTGCGTAAACCGAGCGACATCCAATTCAACAAGAAAATCCTGTTTAGCAACTACACCCCGAAGAAAACCGATTATAGCACCTACCAGCTGAGCTATACCCCGACCGAAATGGAGACCCCGGCGTTCGACGAACTGATGAGCGTTCTGTACGCGGATGAGGAACTGGAGAAGATTCTGTGGGCGCTGGGTGCGCTGCTGAGCGGCAAGATGAGCAAAATCGAAAAGTTTCTGTACCTGTATGGTGCGAAAGGCACCGGCAAGGGCACCGCGATTAACATCATTAAGAAAATGTTCGAGCAGTACTGGGGTCCGATTGACCTGCGTACCCTGACCGGTAGCAGCGAATTTGCGACCAGCGCGGTGACCGAGGTTCCGATGCTGATCGACGCGGATAGCGACATTAGCCGTATCCGTAACGAACAAAACCTGCTGAAACTGACCAGCCACGAGGAACTGATGCGTAACGTGAAGTTCAAAAGCCCGTACCCGGTGGTTTTTGACGGTCTGCTGATCACCGCGAGCAACGAGCGTTATACCGTGAAGAACAAAGATAGCGGCATTACCCGTCGTGCGATCGTGGTTAACCCGACCGGCAAGACCGTTCCGTATGACATGTACCAGCGTCTGTACAAGCAAATCGATTACGAAATCCCGGGCATCGCGTATCGTGCGATGCAGATCTTCAAGAAACGTGGTGCGAGCTACTATGAGAACCTGGTGGACACCGAAATGGTTGAGTACAGCGATAAGGTTTTCGCGTTTATTCGTGAAAGCAGCTTTAACTACATCGGTAAAGAGTATGTGAAGCTGGTTGACGTGGTTCCGGCGTATCAACGTTACCTGACCGATATGGGTTGGGACAACAAAGGCGCGAAGCGTGAACTGAAGAACGAGCTGCACAAATATTTCGAAAGCTACGTGAAAGATACCAAGGACGAGGAAGGCAACCGTATTTATGACGTTTACAAGGGTTTCCGTACCGATATCGCGTTTCCGGAGGAAGTGAACAACAAAGAAGTTGAGCTGCCGACCCTGGAAATCAACATTGAGGGTCCGGGCGCGTTTGACAAAGAAGCGGCGAAGTATCCGGCGCAGTACGCGAACGAGGCGGGTTATCCGATCAAGAAATGGGACGATGTGCTGGAAACCCTGGATACCCTGGACCCGACCAAACTGCACTACGTGCGTGTTCCGCAAAACCACATCGTTCTGGATTTCGACCTGAAGGACCCGGCGACCGGCGAGAAGAGCTTTGAGATGAACCTGGCGAAGGCGAGCAGCTATCCGCCGACCTACGCGGAGCTGAGCAAAAGCGGTGGCGGTATTCACCTGCACTACATCTATGATGGTGACGTGAGCAAGCTGGCGCCGATCATTGAAGACGATATCGAGATTAAGGTTTATAGCGGCAAAAGCAGCCTGCGTCGTAAACTGAGCCTGCGTAACGACCTGCCGATTGCGCACATCAGCACCGGTCTGCCGTTCAAAGAGGAAAAGGAGACCCTGTACAAGGATATTGAAAACATCGTGTGGACCGAGACCAAACTGCGTGCGTTCATCGAAGCGGCGATGAACAAGGAGCACCACGGTGCGACCAAACCGGAAATTGACTTTATCAACGCGAAGCTGAACGAGGCGAAAGACACCGGCGTGCAGTATGATCTGAGCAGCATGCGTATGGCGGTTCTGAAGTTCGCGATGACCAGCAGCAACCAGAGCGAATACTGCATTAAACTGGTTAGCCAAATGCCGTTTAGCACCATCGAAACCGAGGAAGAGCTGAACCAGAGCGAGATGATTGTGGACGATAAAGATATTACCTTCTACGACATCGAGGTTTTTCCGAACCTGCTGCTGATCTGCTGGAAGAAATACGGTCAACCGGGCGTGATTTGGTATAACCCGACCCCGGAAAAGATCACCGAGCTGATGCGTCACAACCTGGTTGGTTTCAACAACCGTAAATACGACAACCACATCATTTATAACCGTATGCTGGGCGCGAGCAACCTGGATATGTACCACCAGAGCCAGCAAATCATTAACCAGCAAATGAGCGCGATGATGCAACCGGCGTATGGTATTAGCTACGCGGACCTGTATGAAATGATGGATATCAAGCAGAGCCTGAAGAAATGGGAAATTGAGCTGGGCATCAAACACGACGAACTGGAGTTCCCGTGGGACCAACCGCTGCCGGAAGATGAGTGGGCGCGTTGCGCGGAGTATTGCATGAACGATGTGATTGCGACCGAAGAGCTGTTCAAGAGCAGCGCGGGTCAGGATGCGTACACCGCGCGTAAAATCCTGTGCGAACTGACCGATATGCCGGTGAACTTTAAGACCCAGACCCTGGCGGAGAAATTCCTGTTTGGCGACGATCCGCGTCCGCAAGACAAGTTCGTTTGGTACGATCTGGCGAAAGAATTTCCGGGTTACAAGTATAGCTACGGCAAAAGCGATTATCTGGGTGAAGACCCGAGCGAGGGCGGTTATGTGTACAGCGAACCGGGTGTTTACAAGGATATCTGGCTGCTGGATGTGGAGAGCCTGCACCCGCACAGCCTGATCGCGATTAACTACTTCGGCCCGTATACCCCGAAGTTTGCGGCGCTGGTTAAATGCCGTATGCACATTAAGCACAAAGAGTTCGACCTGGCGGCGCACGCGTTTGATGAAATTGACCCGGAGCTGAGCCGTAAGCTGGCGCCGTTCCTGAAAGATCCGAGCCAGGCGGGCGGTCTGGGTCATGCGATGAAAATCGTGATTAACATCGTTTACGGTATGACCAGCGCGAAATATGATAACAAGTTCCGTGACCCGCGTAACGTGGATAACATCGTTGCGAAGCGTGGCGCGCTGTTTATGATGATGCTGAAACGTGAGCTGCAGGCGATGGGTCAGCAAGTGGTTCACGTTAAGACCGACAGCTTTAAACTGCCGGGCGCGAACGAAAAGATCTATGAGTACTGCCAAAAACGTGCGCACGAATTCGGTTACAACTTTGATCACGAGGCGACCTTCAGCCGTCTGGCGCTGGTGAACAAGGCGGTTATCATTGGCGAATTTGCGTATCCGGAGTACAAAGCGGGTGAATGGGAGCCGACCGGTGCGCAGTTCGCGTTTCCGTATGTGTACAAGAAACTGTTCAGCGGTGAAGACCTGCTGGAGGACGATTTTGCGATCCTGAAAAGCGTTAAGAGCAGCATTCTGCTGGGTGATCGTTTCATCGGCAAGAACGCGCAATTTTATGCGAGCAAAACCGGCGAAGACCTGTTCCGTACCGGTGAGGTGGATCTGGCGAAGAAAGTGCAGACCCGTGTTAACGGCCAAATGAAGAAAGATCCGGACGGTTACATTGACATCACCAAAATTGCGAAGGAACTGAACATCACCGATGAAGAGGTGGGTGAGATTATGGAGAGCGGTTTCAAGCCGAAAATGGTTGAGACCCGTAACAGCATCAGCGGTACCAAAGGCTTTAAGTGGAACCTGTGGAGCGACTACAAGGGTAAAGAGGATATTGACGTGAAGTACTATGAAGATCTGGTGGAGGACGCGGTTAAAAACATCTACAGCGTTGGTGATGGCGACATCATTTTCAAAGGCACCAAGTGGGAAGGTTATGGCAACCACGAGCTGGTTTAA |
| --- |
| **The amino acid sequence of the full-length GP55:**  MDFYKTLDERVTNRNASSDYVIWPDFKYAGAKDIVVKGGDFYAYWNGETWITDIDSLIKAIDQDSWERFNELRALDPNLRINVKQMDFASSGIMAKFQQYCQLRKPSDIQFNKKILFSNYTPKKTDYSTYQLSYTPTEMETPAFDELMSVLYADEELEKILWALGALLSGKMSKIEKFLYLYGAKGTGKGTAINIIKKMFEQYWGPIDLRTLTGSSEFATSAVTEVPMLIDADSDISRIRNEQNLLKLTSHEELMRNVKFKSPYPVVFDGLLITASNERYTVKNKDSGITRRAIVVNPTGKTVPYDMYQRLYKQIDYEIPGIAYRAMQIFKKRGASYYENLVDTEMVEYSDKVFAFIRESSFNYIGKEYVKLVDVVPAYQRYLTDMGWDNKGAKRELKNELHKYFESYVKDTKDEEGNRIYDVYKGFRTDIAFPEEVNNKEVELPTLEINIEGPGAFDKEAAKYPAQYANEAGYPIKKWDDVLETLDTLDPTKLHYVRVPQNHIVLDFDLKDPATGEKSFEMNLAKASSYPPTYAELSKSGGGIHLHYIYDGDVSKLAPIIEDDIEIKVYSGKSSLRRKLSLRNDLPIAHISTGLPFKEEKETLYKDIENIVWTETKLRAFIEAAMNKEHHGATKPEIDFINAKLNEAKDTGVQYDLSSMRMAVLKFAMTSSNQSEYCIKLVSQMPFSTIETEEELNQSEMIVDDKDITFYDIEVFPNLLLICWKKYGQPGVIWYNPTPEKITELMRHNLVGFNNRKYDNHIIYNRMLGASNLDMYHQSQQIINQQMSAMMQPAYGISYADLYEMMDIKQSLKKWEIELGIKHDELEFPWDQPLPEDEWARCAEYCMNDVIATEELFKSSAGQDAYTARKILCELTDMPVNFKTQTLAEKFLFGDDPRPQDKFVWYDLAKEFPGYKYSYGKSDYLGEDPSEGGYVYSEPGVYKDIWLLDVESLHPHSLIAINYFGPYTPKFAALVKCRMHIKHKEFDLAAHAFDEIDPELSRKLAPFLKDPSQAGGLGHAMKIVINIVYGMTSAKYDNKFRDPRNVDNIVAKRGALFMMMLKRELQAMGQQVVHVKTDSFKLPGANEKIYEYCQKRAHEFGYNFDHEATFSRLALVNKAVIIGEFAYPEYKAGEWEPTGAQFAFPYVYKKLFSGEDLLEDDFAILKSVKSSILLGDRFIGKNAQFYASKTGEDLFRTGEVDLAKKVQTRVNGQMKKDPDGYIDITKIAKELNITDEEVGEIMESGFKPKMVETRNSISGTKGFKWNLWSDYKGKEDIDVKYYEDLVEDAVKNIYSVGDGDIIFKGTKWEGYGNHELV |
